# Patient sex does not affect endoscopic outcomes of biologicals in inflammatory bowel disease but is associated with adverse events

**DOI:** 10.1007/s00384-020-03663-2

**Published:** 2020-06-26

**Authors:** Mitchell R. K. L. Lie, Emma Paulides, C. Janneke van der Woude

**Affiliations:** Department of Gastroenterology and Hepatology, Erasmus MC, Rotterdam, The Netherlands

**Keywords:** Inflammatory bowel disease, Sex differences, Biologicals

## Abstract

**Purpose:**

Biological therapies are currently the mainstay in the treatment of patients with inflammatory bowel diseases (IBD). Several factors are known to influence the efficacy and tolerability of biologicals, such as CRP levels or previous biological use. Whether patient sex affects the efficacy or tolerability is unclear but would help with better risk and benefit stratification. This systematic review assesses patient sex on the efficacy and tolerability of biological therapies in IBD patients.

**Methods:**

A systematic literature review was performed using Embase (including MEDLINE), MEDLINE OvidSP, Cochrane Central Register of Controlled Trials, Web of Science and PubMed. The primary outcome was the influence of patient sex on endoscopic outcomes in IBD patients treated with biologicals. The secondary outcome was the influence of patient sex on adverse events. Studies were included in the assessment regardless of study type or setting.

**Results:**

The search yielded 19,461 citations; after review, 55 studies were included in the study, involving 28,465 patients treated with adalimumab, certolizumab pegol, infliximab, or vedolizumab. There was no significant association between patient sex and endoscopic efficacy in 41 relevant studies. Increased adverse events were associated with female sex in 7 out of 14 relevant studies.

**Conclusions:**

There is no evidence for a sex difference in endoscopically measured response to biological therapies in IBD patients. However, there is an influence of sex on the occurrence of adverse events.

**Electronic supplementary material:**

The online version of this article (10.1007/s00384-020-03663-2) contains supplementary material, which is available to authorized users.

## Introduction

Due to their chronic nature, inflammatory bowel diseases (IBD), consisting of Crohn’s disease (CD) and ulcerative colitis (UC), usually require life-long drug therapies. The treatment paradigm seems to switch, and the current approach has been changed to a more accelerated step-up management of the IBD patient. Currently, a large proportion of IBD patients are treated with biologicals, with studies reporting in the range of 20–25% in Western countries [1–3], and the use of biologicals seems to increase [3, 4]. This increasing use necessitates the identification of factors predictive of drug efficacy and drug survival. Previously identified factors known to affect efficacy and tolerability of biological therapies in IBD patients include previous use of another biological drug [5], baseline C-reactive protein levels [6] and serum drug levels [7]. A simple factor to include in the treatment strategy could be patients’ sex. Sex is already implicated as an important factor in the pathogenesis of IBD [8].

However, the current evidence on the role of patient sex on the actual response to biological therapies is conflicting. Several studies specifically report on differences in response and adverse events between male and female IBD patients treated with biologicals [9, 10] whereas other studies report no significant differences between male and female patients [11, 12]. Thus, it remains unclear if a patients’ sex plays a role in the efficacy or tolerability of biological therapies. This study aimed to systematically search the literature for evidence regarding the possible association of patient sex and biological therapies, concerning efficacy (measured objectively via endoscopy) and the occurrence of adverse events.

## Objectives

This study aims to systematically review the literature for studies concerning established biological therapies for patients with inflammatory bowel disease, examining the possible influence of patient sex on:

- Objectively measured efficacy, defined as disease activity measured via endoscopy. Examples of this primary outcome include sigmoidoscopy, ileocolonoscopy and capsule endoscopy.
- Adverse events defined as any adverse event possibly related to biological use. Examples of this secondary outcome are infusion reactions, injection site reactions and hypersensitivity reactions.

## Methods

### Search strategy

A systematic database search was performed on 08 April 2019, without restrictions on language, publication year or publication status. The search was performed by librarians specialised in database searches. The search was performed in the following databases: Embase (including MEDLINE), MEDLINE OvidSP, Cochrane Central Register of Controlled Trials, Web of Science and PubMed. The detailed digital search strategy is provided in the Supplemental material, Appendix 1. Additionally, the reference lists of all potentially relevant articles were studied for further trials. Any studies found trough this search also had their reference lists studied.

### Review and study selection process

Titles and abstracts identified through the search strategy were assessed by two independent reviewers (ML and EP) for potential eligibility, using pre-defined criteria as described in Supplemental material, Appendix 2. Disagreements were settled in consensus and, if necessary, after discussion with a third independent reviewer (CW). The manuscripts deemed potentially eligible for inclusion were obtained for full text review. The full texts were assessed by the two independent reviewers, using pre-defined eligibility criteria as described in Supplemental material, Appendix 3. Discussions with the third independent reviewer were used to resolve disagreements.

### Data extraction

Data from the eligible studies was extracted using a standardised form by the two primary reviewers. Differences in the extracted data were resolved through consensus or, if necessary, discussion with the third independent reviewer. For each study, the following data was extracted:

1. Study type and methods (including study duration, loss to follow-up)
2. Participants (including age, disease type, duration of treatment prior to enrolment)
3. Interventions (including drug, dosage, duration, formulation)
4. Outcomes (including definitions of the primary and secondary outcomes)

### Quality assessment

The risk of bias of included studies was assessed using either the Newcastle-Ottawa Scale (NOS) for cohort studies [13] or the Cochrane risk of bias assessment tool for randomised controlled trials (RCT) and post hoc analyses of RCTs [14]. The NOS ranges from 0 to 9, with 9 resembling the best score and the lowest risk of bias. The Cochrane tool assigns low risk, unclear risk or high risk to randomisation, allocation and reporting bias, respectively. The assessments were performed by the two primary reviewers, and in case of disagreement, consensus was found after discussion with the third reviewer.

### Data synthesis and statistical analysis

Results are reported using the summary measure provided by the included studies (e.g. odds ratio (OR), hazard ratio (HR), difference in means) with the respective *P* values and/or confidence intervals. If only proportions were reported, the OR was calculated.

For meta-analysis, where applicable, studies were pooled using a random-effects model, regardless of statistical heterogeneity. Heterogeneity was tested using the Chi-squared test, the *I*-squared test and visual inspection of forest plots. If heterogeneity was present, we attempted to investigate the cause thereof (such as methodological factors or the outcome assessment). In the case of high heterogeneity (*I*^2^ > 75%), studies were pooled only if the direction of their results was consistent. Subgroup analysis or meta-regression would be performed post hoc, if sufficient studies were included for meta-analysis.

## Results

### Results of the search

**Fig. 1:**
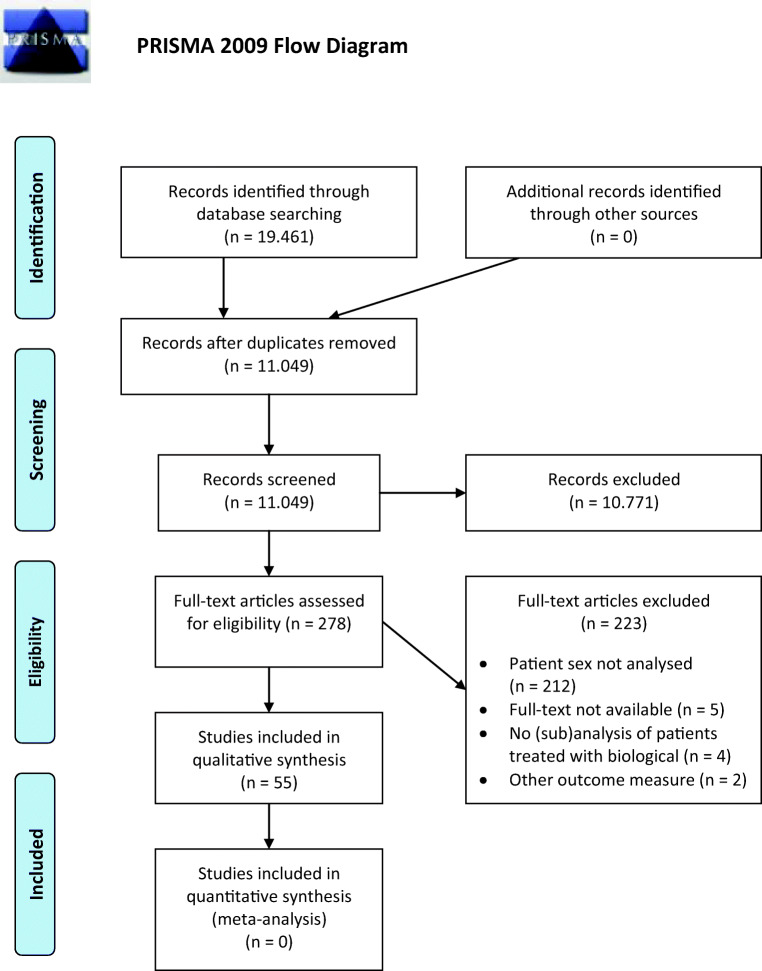
PRISMA flowchart of identification and selection of studies

The literature search performed on 08 April 2019 identified 19,461 citations, of which 11,049 remained after automatic removal of double entries (Fig. 1). After reviewing title and abstracts, 10,771 manuscripts were considered irrelevant (e.g. did not study biological, case reports, abstract format only, in vitro study, see also Supplemental Table 1). This resulted in 278 potentially relevant studies. Examining the reference lists did not yield additional potentially useful manuscripts. In total, 273 manuscripts were assessed completely for eligibility as 5 manuscripts could not be retrieved (Fig. 1, flowchart). Of these 273 studies, 217 were excluded for various reasons (Supplemental Table 2). The remaining 55 studies were included in this review (Tables 1 and 2) [7, 9, 15–67].

**Table 1:** Characteristics of included studies concerning patient sex and endoscopic efficacy

Biological	Study type	Patients	Author (ref)	Outcome, measurement time point	Patient sex associated with outcome?
ADA, induction of remission	Prospective	43 CD	Hall [37]	CECDAI, 52 weeks	Not associated
	Retrospective	201 UC	Kiss [43]	MH, 12 months	Not associated
	Retrospective	43 UC	Papamichael [7]	MH, 8–14 weeks	Not associated
	Retrospective	77 CD	Rismo [58]	MH, variable time-point	Not associated
	RCT post-hoc	135 CD	Watanabe [65]	MH, 26 and 52 weeks	Not associated
ADA, maintenance of remission	Cross-sectional	98 IBD	Juncadella [40]	CD: MH; UC: endoscopic Mayo ≤ 1	Not associated
	Cross-sectional	40 IBD	Roblin [59]	CD: MH; UC: endoscopic Mayo ≤ 1	Not associated
	Cross-sectional	60 CD	Zittan [67]	MH	Not associated
ADA, post-operative	RCT post-hoc	101 CD	de Cruz [26]	Disease recurrence, 6 months	Not associated
	RCT post-hoc	84 CD	Taxonera [5]	Disease recurrence, 52 weeks	Not associated
IFX, induction of remission	Prospective	285 UC	Arias [15]	MH, 10–14 weeks	Not associated
	Combined^a^	126 UC	Armuzzi [17]	MH, 12 weeks and 12 months	Not associated
	RCT post-hoc	508 CD	Bouguen [19]	MH, 26 weeks	Not associated
	Prospective	30 UC	Brandse [20]	Endoscopic Mayo decrease ≥ 1 and 8 weeks	Not associated
	Prospective	63 UC	Farkas [30]	MH, 14 weeks	Not associated
	Prospective	44 UC	Hassan [38]	MH, 12 weeks	Not associated
	Retrospective	42 UC	Kelly [41]	MH, 48 weeks	Not associated
	Retrospective	101 UC	Papamichael [7]	MH, 10–14 weeks	Not associated
	Retrospective	49 UC	Ribaldone [56]	Total Mayo decrease ≥ 3, 6 months	Not associated
	Retrospective	49 UC	Rismo [57]	Endoscopic Mayo ≤ 1, 8–12 weeks	Not associated
	Retrospective	97 CD	Shen [61]	MH, 10 weeks	Not associated
	Retrospective	126 CD	Thomas [63]	Complete/near-complete MH, 12–20 weeks	Not associated
IFX, maintenance of remission	Retrospective	271 IBD	Kelly [42]	CD: SES-CD < 3; UC: endoscopic Mayo ≤ 1	Not associated
	Prospective	35 CD	Koga [44]	MH	Not associated
	Retrospective	110 CD	Papamichael [53]	MH	Not associated
	Prospective	54 IBD	Paul [54]	MH	Not associated
VED, induction of remission	Retrospective	48 CD	Crowell [24]	Undefined endoscopic improvement, 45 weeks	Not associated
	Retrospective	179 IBD	Dreesen [27]	CD: MH, 22 weeks; UC: endoscopic Mayo ≤ 1, 14 weeks	Not associated
	Retrospective	212 CD	Dulai [29]	MH, 6 and 12 months	Not associated
	Retrospective	222 IBD	Kotze [45]	CD: MH or radiographic remission, 3, 6 and 12 months; UC: endoscopic Mayo = 0, 3, 6 and 12 months	Not associated
	Retrospective	321 UC	Narula [50]	Endoscopic Mayo = 0 and 12 months	Not associated
	Prospective	82 IBD	Yacoub [66]	CD: MH or radiographic remission, 12 months; UC: endoscopic Mayo ≤ 1, 12 months	Not associated
ADA, IFX, remission induction	Retrospective	248 IBD	Beigel [18]	CD: SES-CD = 0; UC: endoscopic Mayo = 0; for both groups after median 11–25 months	Not associated
	Retrospective	48 UC	Dahlen [25]	Total Mayo decrease ≥ 3, 14 weeks	Not associated
	Prospective	50 CD	Kuzela [46]	Normal mucosal appearance via capsule endoscopy, 1 year	Not associated
	Retrospective	107 CD	Papaconstantinou [51]	MH, 12–20 weeks	No associated
ADA, IFX, maintenance of remission	Retrospective	64 UC	Morita [48]	UCEIS 0/0/0 or 1/0/0	Not associated
	Retrospective	145 IBD	Ungar [64]	CD: SES-CD < 3; UC: endoscopic Mayo ≤ 1	Not associated
ADA, IFX, post-operative	Retrospective	73 CD	Fay [31]	Disease recurrence, after median 15 months	Not associated
	Retrospective	36 CD	Hiraoka [39]	Disease recurrence, time not specified	Not associated
	Retrospective	44 CD	Preda [55]	Disease recurrence, time not specified	Not associated
ADA, CZP, IFX, remission induction	Prospective	69 IBD	Guidi [36]	CD: CDEIS < 3, 1 year; UC: endoscopic Mayo ≤ 1, 1 year	Not associated

Grouped by biological studied

Abbreviations: *ADA*, adalimumab; *CD*, Crohn’s disease; *CDEIS*, Crohn’s disease endoscopic index of severity; *CECDAI*, capsule endoscopy Crohn’s disease activity index; *CZP*, certolizumab pegol; *IBD*, inflammatory bowel disease; *IFX*, infliximab; *MH*, mucosal healing; *RCT*, randomised controlled trial; *SESCD*, simple endoscopic score for Crohn’s disease; *UC*, ulcerative colitis; *VED*, vedolizumab

^a^Combined retrospective and prospective cohort

**Table 2:** Characteristics of included studies concerning patient sex and adverse events. Grouped by biological studied.

Biological	Study type	Patients	Author (ref)	Outcome	Patient sex associated with outcome?
ADA, induction of remission	Retrospective	188 CD	Lie [47]	Any adverse event	More often in female patients (OR, 1.27)
				Treatment withdrawal due to adverse events	More often in female patients (OR, 1.93)
	Retrospective	5345 IBD	Colombel [23]	Death (standardised mortality ratio)	Lower in male UC patients (ratio, 0.38)
IFX, induction of remission	Prospective	810 IBD	Armuzzi [16]	Serious adverse events	More often in female patients (HR, 1.96)
	Retrospective	743 IBD	Fidder [33]	Serum sickness-like disease, skin lesions	More often in female patients (OR, 3.74 and OR, 1.90)
				Mortality, neoplasia, serious infections, infusion reactions, auto-immune phenomena	Not associated
	Retrospective	336 IBD	Mourad [49]	Any adverse event	Not associated
IFX, maintenance of remission	Retrospective	512 CD	Colombel [21]	Serious infections	Not associated
	Retrospective	3161^a^	Ducharme [28]	Any acute adverse drug reaction within 24 h of IFX infusion	More often in female patients (OR, 1.54)
	Retrospective	169 CD	Gonzales (2017)	Infusion reactions	Not associated
	Retrospective	197 IBD	Greener [35]	Infections	Not associated
	Retrospective	100 IBD	Seiderer [60]	Any adverse event	Not associated
VED, induction of remission	RCT post hoc	2884 IBD	Colombel [22]	Any serious infection	Not associated
	RCT post hoc	2243 IBD	Feagan [32]	Lower respiratory tract infection	More often in female patients (HR, 2.11)
				Upper respiratory tract infection	Not associated
ADA, IFX, remission induction	Retrospective	149 CD	Teriaky [62]	Any adverse event	Not associated
ADA, IFX, maintenance of remission	Retrospective	843 IBD^b^	Zelinkova [9]	Any adverse drug reaction	More often in female patients (OR, 2.21)
				Treatment withdrawal due to adverse events	More often in female patients (OR, 2.46)

Abbreviations: *ADA*, adalimumab; *CD*, Crohn’s disease; *CZP*, certolizumab pegol; *HR*, hazard ratio; *IBD*, inflammatory bowel disease; *IFX*, infliximab; *OR*, odds ratio; *RCT*, randomised controlled trial; *UC*, ulcerative colitis; *VED*, vedolizumab

^a^Of whom, 1936 Crohn or ulcerative colitis

^b^Of whom, 150 used biologicals

### Meta-analysis

Several studies employed similar outcome measures (e.g. post-operative recurrence [31, 39, 55] or mucosal healing after 1 year [29, 45, 66]) and were thus suitable for meta-analysis. However, the studies in both the primary and the secondary outcomes did not report exact summary measures or the frequencies in which the outcomes of interest occurred in male and female patients. Therefore, the studies were reviewed systematically but no meta-analysis could be performed.

### Primary outcome

In total, 41 studies were included studying the objectively measured efficacy of biologicals in 4736 patients [7, 15, 17–20, 24–27, 29–31, 36–46, 48, 50–59, 61, 63–67]. Concerning methodology, 24 studies were retrospective [7, 18, 24, 25, 27, 29, 31, 39, 41–43, 45, 48, 50–53, 55–58, 61, 63, 64], 10 were prospective cohorts [15, 20, 30, 36–38, 44, 46, 54, 66], 3 were post hoc analyses of RCTs [17, 26, 65], 3 were cross-sectional [40, 59, 67] and 1 study was a combination of a retrospective and prospective cohort [17] (Table 1).

The quality of the cohort studies was fair to good, with a median NOS of 7 (range 4–8), the risk of bias for the post hoc studies was considered unclear (Supplemental Tables 3a and 3b). Regarding the post hoc studies, the study by Bouguen et al. [19] involves a RCT with low risk of bias; however, the post hoc nature increases the risk of reporting bias. Additionally, this study used only a subset of the RCT population, creating an unclear risk of selection bias. The study by de Cruz et al. [26] involved an open-label RCT, as such there is risk of allocation and performance bias; however, the risk of detection bias was low as the endoscopic outcome was evaluated by blinded central readers. The post hoc analyses by Watanabe et al. [65] was also based on an open-label RCT; therefore, the study was at risk of allocation, performance and detection bias.

### Studies examining one biological

Thirty studies examined only one biological [7, 15, 19, 20, 24, 26, 27, 29, 30, 37, 38, 40–45, 50, 52–54, 56–59, 61, 63, 65–67], 9 studied adalimumab [26, 37, 40, 43, 52, 58, 59, 65, 67], 16 studied infliximab [7, 15, 17, 19, 20, 30, 38, 41, 42, 44, 53, 54, 56, 57, 61, 63] and 6 studied vedolizumab [24, 27, 29, 45, 50, 66]. The details concerning setting (e.g. retrospective, prospective), use (i.e. for induction, maintenance or post-operative prophylaxis), patients (e.g. CD or UC) and outcome measures (e.g. endoscopic remission) varied widely.

### Adalimumab

There were considerable differences in study settings and methodologies in the nine studies concerning adalimumab. Three studies were cross-sectional [40, 59, 67], three were retrospective cohorts [43, 52, 58], two were post-hoc studies [26, 65] and the last study examined a prospective cohort [37]. Nevertheless, all studies found that patient sex was not significantly associated with endoscopic outcomes, measured at variable time points (e.g. mucosal healing after 8–14 weeks [52] or mucosal healing after 1 year [65]).

### Infliximab

Similar to the adalimumab studies, the 16 infliximab studies were varied in setting, scope and statistical methods. Of these studies, Papamichael et al. [7] found in univariable analysis that female UC patients were significantly more likely to achieve mucosal healing, measured 10–14 weeks after start of infliximab. However, this effect was no longer statistically significant in the corrected multivariable analysis. Similarly, all other infliximab studies found no significant association between patient sex and endoscopic outcomes, regardless of the statistical method employed.

### Vedolizumab

The six studies examining patients using vedolizumab were more homogenous than the adalimumab or infliximab studies. Five of the vedolizumab studies were retrospective [24, 27, 29, 45, 50], and all six studies examined vedolizumab as remission induction. In the only prospective study by Yacoub et al. [66], in univariable analysis, female IBD patients were significantly more likely to achieve mucosal healing after 1 year than male IBD patients; however, in the corrected multivariable analysis, the difference between male and female patients was no longer statistically significant. The other vedolizumab studies also found no significant associations between patient sex and endoscopic outcomes.

### Studies examining multiple biologicals

Of the included studies involving multiple biologicals, seven examined a population treated with adalimumab or infliximab [15, 25, 31, 39, 46, 48, 51, 55, 64] and one concerned IBD patients treated with adalimumab, certolizumab or infliximab [36]. The first group of studies were all of a retrospective nature, with varying populations of CD patients, UC patients or both, as described in Table 2. The study concerning adalimumab, certolizumab or infliximab examined a prospective cohort of IBD patients.

### Adalimumab or infliximab

Seven studies examined combined groups of patients, either treated with adalimumab or infliximab. All seven studies were retrospective but in varied patient groups and settings. None of the studies found a relation between endoscopic outcomes and the use of adalimumab or infliximab.

### Adalimumab, certolizumab pegol or infliximab

Guidi et al. [36] assessed a prospective cohort of IBD patients treated with adalimumab, certolizumab pegol or infliximab for remission induction. Via logistic regression, no association was found between mucosal healing after 1 year and patient sex.

### Secondary outcome

In total, 14 studies were included, assessing 17,680 patients treated with biologicals [9, 16, 21–23, 28, 32–35, 47, 49, 60, 62]. Ten studies were retrospective [9, 21, 28, 33–35, 47, 49, 60, 62], one was prospective [16] and the remaining 3 were post hoc analyses of RCTs [22, 23, 32] (Table 2).

The quality of the different studies was poor, with a median NOS of 5 (range 5–8). The three post hoc studies were considered of low-risk of bias, as the original RCTs were of low risk themselves and the safety analyses were pre-specified and used the whole study population (Supplemental Tables 3a and 3b).

### Studies examining one biological

In total, 12 studies consisted of cohorts concerning a single biological [16, 21–23, 28, 32–35, 47, 49, 60]. Two studies involved adalimumab [23, 47], eight involved infliximab [16, 21, 28, 33–35, 49, 60] and two assessed vedolizumab [22, 32]. Of the adalimumab studies, one consisted of a cohort of CD patients [47] and the other of a cohort of IBD patients [23]. For infliximab, seven studies were retrospective cohorts [21, 28, 33–35, 49, 60] and one was prospective [16]. The study populations consisted of CD patients in two studies [21, 34] and IBD patients in six studies [16, 33–35, 49, 60]. The remaining infliximab study involved mostly IBD patients but also included patients that used infliximab for rheumatologic or dermatologic diseases [28]. The two vedolizumab studies were both post hoc analyses of IBD patients treated with vedolizumab.

### Adalimumab

Two studies were identified that examined patient sex and adverse events during adalimumab use. In a retrospective cohort of CD patients treated with adalimumab for remission induction, Lie et al. [47] described an increased frequency of adverse events reported by female patients compared with male patients (OR, 1.27; *P* < 0.01). Additionally, female patients reported adverse events as a reason for stopping adalimumab more often than male patients (OR, 1.93; *P* = 0.02).

In a large post hoc analysis of 16 RCTs and their open label extensions involving 5345 IBD patients, Colombel et al. [23] calculated standardised mortality ratios and compared these with an age- and sex-matched control group. In this comparison, the standardised mortality ratio of male UC patients was lower compared with matched controls (ratio, 0.38), but no statistically significant difference was found for female UC patients or male or female CD patients.

### Infliximab

Eight studies described adverse events during infliximab use and patient sex. Three studies found significant associations, with Armuzzi et al. [16] describing a prospective cohort of 810 Italian IBD patients who started treatment with the infliximab biosimilar CT-P13, both for remission induction and for maintenance of remission. In this cohort serious adverse events occurred less frequent in male IBD patients than IBD female patients (HR, 0.51; CI, 0.35–0.76; *P* = 0.001). In a large retrospective study involving 3161 patients treated with infliximab, Ducharme et al. [28] examined adverse events. However, in this large cohort, 55% of patients received infliximab because of IBD, but the remaining 45% were treated with infliximab because of rheumatologic or dermatologic conditions. Nevertheless, within this heterogeneous group of diseases, an acute drug reaction (i.e. and adverse event within 24 h of the infliximab infusion) was more likely to occur in female patients than in male patients (OR, 1.54; *P* < 0.001). Unfortunately, no sub-analysis was performed to assess if this association remains in only IBD patients. Fidder et al. [33] retrospectively compared a cohort of 743 IBD patients treated with infliximab for remission induction with 666 IBD patients without exposure to biologicals. Serum sickness-like disease occurred more frequently in female patients than in male patients (OR, 3.74; *P* < 0.01). Skin lesions were also reported more often in female patients than in male patients (OR, 1.90; *P* < 0.01). However, no sex difference could be detected for mortality, neoplasia, serious infections, infusion reactions and auto-immune phenomena. The five other studies found no association between patient sex and adverse events during infliximab use.

### Vedolizumab

Two studies examined the possible role of patient sex on the occurrence of adverse events during vedolizumab therapy. In a post hoc analysis of the GEMINI-1, GEMINI-2 and GEMINI open-label extension trials, Feagan et al. [32] examined the occurrence of respiratory tract infections in IBD patients treated with vedolizumab. They found that lower respiratory tract infections are more likely to occur in female patients than in male patients (HR, 2.11; *P* = 0.03). This effect was only seen in UC patients, not in CD patients. Furthermore, no association between patient sex and upper respiratory tract infections was found. A general analysis of safety of vedolizumab was performed by Colombel et al. [22] using post hoc analysis of data from the GEMINI-1, GEMINI-2, GEMINI-3 and GEMINI open-label extension trials. In this study, patient sex was not found to be a significant risk factor for the occurrence of serious infections. Patient sex was not studied in analyses of other types of adverse events.

### Studies examining multiple biologicals

#### Adalimumab or infliximab

In total, two studies were identified that examined the role of patient sex on adverse events during the use of adalimumab or infliximab [9, 62]. One study found a significant association between patient sex and adverse events. Zelinkova et al. [9] examined adverse events in a retrospective cohort of 843 IBD patients. In separate analyses of 150 patients treated with adalimumab or infliximab, adverse drug reactions were found to occur significantly more frequently in female patients than in male patients (OR, 2.21; *P* = 0.01). Further sub-analyses per drug revealed similar associations, though the association in adalimumab users was not statistically significant, possibly due to low patient numbers. Of note, this study also found that female patients stopped anti-TNF treatment more often than male patients due to adverse drug reactions (OR, 2.46).

The other study by Teriaky et al. [62] also examined a cohort of CD patients treated with adalimumab or infliximab but found no association between the patient’s sex and the occurrence of adverse events.

## Discussion

The objectives of this review were to assess the possible influence of patient sex on biological therapies, on endoscopic outcomes and adverse events in the treatment of inflammatory bowel disease. To our knowledge, this was the first systematic review investigating this research question. With regard to efficacy, none of the studies found an association between patient sex and endoscopically measured efficacy of biological therapies. As for adverse events, half of the included studies found an association between patient sex and various adverse events, with all these studies suggesting that these events occur more frequently in female patients.

The intention of this study was to perform a meta-analysis of the included studies; however, several factors precluded synthesis of the data via meta-analysis. Firstly, outcome measures varied amongst studies, with the definitions of adverse events varying from ‘any adverse reaction’ to ‘severe infections’. Secondly, the time-point at which outcomes were measured differed amongst the studies. Thirdly, the study populations were heterogeneous, with some studies examining biological naïve patients and others biological experienced patients or post-operative patients. Fourthly and most importantly, many studies simply reported that patient sex was not associated with the studied outcome, but without providing exact summary measures (e.g. odds ratio, difference in means) or the exact frequencies in which the outcome occurred in male and female patients, respectively. This prevented us from calculating summary measures to perform meta-analysis.

Pharmacokinetic studies in IBD patients concerning infliximab [68, 69] and vedolizumab [70] reported a sex difference regarding clearance and distribution volume. Similarly, in adalimumab, a sex difference for apparent clearance has been reported in rheumatoid arthritis patients [71], but the kinetics have not yet been studied in IBD patients. Based on these preliminary studies, it could be hypothesised that sex differences both in efficacy and adverse event rates could be present in IBD patients treated with biologicals.

However, we found no evidence for a sex difference in objectively measured endoscopic disease outcomes. This strongly suggests that biological therapies are effective regardless of patient sex, probably because the underlying inflammatory pathways affected by these therapies are not significantly different between female and male IBD patients. The lack of a sex difference in efficacy of biologicals is also seen in rheumatology patients [72, 73] and dermatology patients [74, 75] treated with anti-TNF agents.

Nevertheless, there have been consistent reports of a sex difference in IBD patients treated with biologicals, with decreased drug survival (i.e. the proportion of patients still using the drug after a set period of time) in female patients [76, 77]. However, if the efficacy of biologicals is similar in men and women, as shown by this review, this strongly suggests that factors other than primary non-response are responsible for the decreased drug survival. In populations that were not treated with biologicals, literature suggests increased rates of adverse events in females. In a large safety analysis of seven observational studies (none in IBD patients), female sex was associated with the increased occurrence of side effects [78]. A similar result was found in a study regarding hospital admissions [79], wherein female patients were significantly more frequently admitted due to adverse drug reactions than male patients. Therefore, a possible cause of decreased drug survival could be sex differences in adverse events. The results of this systematic review, however, are ambiguous. Though seven studies did find that female sex is associated with adverse events during biological therapy, the other seven included studies found no such association.

This ambiguity is also present in patients treated with biologicals for dermatologic or rheumatologic conditions. For instance, in psoriasis patients some studies reported more adverse events in female patients [80, 81] whereas other studies did not find this association [82]. Similarly, the retention rates of biologicals in psoriasis patients were found to be associated with female sex in some studies [83, 84] but not in others [85, 86]. The same holds true in rheumatology patients treated with biologicals. Several studies reported an association between patient sex and adverse events [87] and drug retention rates [88, 89], whereas other studies found no such association [90].

There are several limitations to this study. Concerning the primary outcome of objectively measured efficacy, the included studies varied greatly in their outcome measures. For instance, in CD patients some studies used Simpe Endoscopic Score for Crohn’s Disease (SES-CD) whilst others used Crohn’s Disease Endoscopic Index of Severity (CDEIS), and in UC patients, some studies used the endoscopic Mayo score whilst others used Ulcerative Colitis Endoscopic Index of Severity (UCEIS). Furthermore, even amongst studies using the same outcome measure, the definitions of response and remission could vary. Additionally, there was great variation in the timing of the endoscopic assessment across the included studies. Though this issue was identified during the review, it was decided to include all studies regardless of the heterogeneity of the outcomes. Though a more stringent set of inclusion criteria regarding endoscopic outcomes would have reduced heterogeneity, it was decided to be as inclusive as possible in order to detect a potential signal concerning sex differences. Furthermore, given the lack of meta-analysable results, using more stringent criteria would not have resulted in a different conclusion.

The issue of high heterogeneity also occurs in the studies included for the adverse event analysis. Similar to the primary outcome, it was decided to use broad inclusion criteria in order to detect a potential signal concerning sex-differences in the occurrence of biological related adverse events. However, of the seven studies that report a sex difference, in three studies the relation between the analysed adverse events and the drug used is debatable. Firstly, Colombel et al. [23] find a lower standardised mortality ratio in male IBD patients treated with ADA, but a direct causal relationship between ADA use and mortality seems unlikely. Similarly, the adverse events analysed by Lie et al. [47] and Armuzzi et al. [16] include not only events probably related to biological use (e.g. injection site reactions, infusion reactions) but also events that are likely unrelated to therapy (e.g. nausea, hair loss, headache). If the analyses in these studies were performed using only adverse events probably related to biological use, the results might no longer be statistically significant. In contrast, the other four studies that identify a significant sex difference specifically analyse events that are possibly therapy related, such as infusion reactions, serum sickness, respiratory tract infections and allergic-type reactions.

In summary, this systematic review finds no evidence for differences in efficacy of biological therapies in female or male IBD patients, as judged endoscopically. Therefore the sex of the IBD patient need not be directly taken into account when considering starting biologicals or optimisation of biological trough levels. The results concerning adverse events are ambiguous, with half of the studies finding an increased occurrence of adverse events in female patients treated with biological therapies, whereas the other half does not. Extra vigilance and proper counselling for treatment emergent adverse events might be warranted. Further investigations of possible sex differences in the occurrence and severity of adverse events could result in more accurate individualised therapy advice and thus improve the quality of personalised medicine.

## Electronic supplementary material

ESM 1 (DOCX 60 kb)
