# Supplementary material for: Patient sex does not affect endoscopic outcomes of biologicals in inflammatory bowel disease but is associated with adverse events
Source: Int J Colorectal Dis. 2020 Jun 26;35(8):1489–500. doi: 10.1007/s00384-020-03663-2 (PMC7340671; doi:10.1007/s00384-020-03663-2)
Supplement: Supplementary file 1 — (DOCX 60 kb) [file 384_2020_3663_MOESM1_ESM.docx]

# Supplementary data

Appendix 1: Electronic database search strategy

Appendix 2: Study criteria for detailed review

Appendix 3: Study criteria for inclusion in review

Appendix 4: References to studies excluded upon detailed review

Appendix 5: MOOSE checklist

Appendix 6: PRISMA checklist

Supplemental Table 1: Studies excluded (with reasons)

Supplemental Table 2: Individual characteristics of excluded studies

Supplemental Table 3a: Quality assessment of cohort studies

Supplemental Table 3b: Risk of bias assessment of randomized controlled trials and post-hoc studies

# Appendix 1: Electronic database search strategy

**Results**

| *Database* | *Number of refs* | *Refs after deduplication* |
| --- | --- | --- |
|  |  |  |
| Embase.com | 6333 | 6185 |
| Medline (Ovid) | 6123 | 1391 |
| Cochrane Central | 15634 | 1003 |
| Web of Science | 5271 | 2388 |
| Google Scholar | 200 | 82 |
|  |  |  |
| **Total** | **19461** | **11049** |

*Deduplicated: 8412*

**Embase.com (Embase incl. Medline): 6333**

**(**'biosimilar agent'/de**/mj** OR 'infliximab'/de**/mj** OR 'ustekinumab'/de**/mj** OR 'vedolizumab'/de**/mj** OR 'adalimumab'/de**/mj** OR 'golimumab'/de**/mj** OR 'certolizumab pegol'/de**/mj** OR (biologicals OR biosimilars OR biologics OR biopharmaceutical* OR ((biologic* OR biosimilar* OR immunomodulat*) NEAR/3 (factor* OR agent* OR drug* OR pharmaceutical*)) OR infliximab* OR Remicade OR ustekinumab* OR stelara OR vedolizumab* OR entyvio OR adalimumab* OR Humira OR golimumab* OR Simponi OR certolizumab* OR Cimzia):ab,ti**)** **AND** **(**'inflammatory bowel disease'/exp**/mj** OR 'enteritis'/de/**mj** OR 'perianal fistulizing crohn disease'/de OR (((inflammatory-bowel) NEAR/3 (disease* OR disorder*)) OR Crohn* OR ileocolitis OR ((regional) NEAR/3 (enterit* OR ileiti*)) OR ((ulcer* OR mucos* OR granulomat*) NEAR/3 (colitis OR enterit*)) OR IBD):ab,ti**)** **AND** ('drug efficacy'/exp OR 'drug effect'/exp OR 'treatment outcome'/exp OR 'biological therapy'/de OR 'therapy effect'/de OR 'treatment response'/de OR (effic* OR effectiv* OR response* OR nonresponse* OR ((treatment* OR therap* OR drug* OR pharmacolog* OR physiologic* OR clinical) NEAR/6 (success* OR effect* OR potential OR outcome* OR failure*))):ab,ti) **NOT** ([animals]/lim NOT [humans]/lim) **NOT (**'Conference Abstract' OR Editorial**)**/it

***Medline (Ovid): 6123***

**(**exp *Biosimilar Pharmaceuticals/ OR (biologicals OR biologics OR biosimilars OR biopharmaceutical* OR ((biologic* OR biosimilar* OR immunomodulat*) ADJ3 (factor* OR agent* OR drug* OR pharmaceutical*)) OR infliximab* OR Remicade OR ustekinumab* OR stelara OR vedolizumab* OR entyvio OR adalimumab* OR Humira OR golimumab* OR Simponi OR certolizumab* OR Cimzia).ab,ti.**)** **AND** **(**exp *Inflammatory Bowel Diseases/ OR *Enteritis/ OR (((inflammatory-bowel) ADJ3 (disease* OR disorder*)) OR Crohn* OR ileocolitis OR ((regional) ADJ3 (enterit* OR ileiti*)) OR ((ulcer* OR mucos* OR granulomat*) ADJ3 (colitis OR enterit*)) OR IBD).ab,ti.**)** **AND** (exp "Physiological Effects of Drugs"/ OR exp Treatment Outcome/ OR Biological Therapy/ OR (effic* OR effectiv* OR response* OR nonresponse* OR ((treatment* OR therap* OR drug* OR pharmacolog* OR physiologic* OR clinical) ADJ6 (success* OR effect* OR potential OR outcome* OR failure*))).ab,ti.) **NOT** (exp animals/ NOT humans/) NOT **(**congresses OR editorial**)**.pt.

***Cochrane Central (trials): 1534***

**(**(biologicals OR biologics OR biosimilars OR biopharmaceutical* OR ((biologic* OR biosimilar* OR immunomodulat*) NEAR/3 (factor* OR agent* OR drug* OR pharmaceutical*)) OR ((monoclonal) NEXT/1 (antibod*)) OR infliximab* OR Remicade OR ustekinumab* OR stelara OR vedolizumab* OR entyvio OR adalimumab* OR Humira OR golimumab* OR Simponi OR certolizumab* OR Cimzia):ab,ti**)** **AND** **(**(((inflammatory-bowel) NEAR/3 (disease* OR disorder*)) OR Crohn* OR ileocolitis OR ((regional) NEAR/3 (enterit* OR ileiti*)) OR ((ulcer* OR mucos* OR granulomat*) NEAR/3 (colitis OR enterit*)) OR IBD):ab,ti**)** **AND** ((effic* OR effectiv* OR response* OR nonresponse* OR ((treatment* OR therap* OR drug* OR pharmacolog* OR physiologic* OR clinical) NEAR/6 (success* OR effect* OR potential OR outcome* OR failure*))):ab,ti)

***Web of Science: 5271***

**TS=((**(biologicals OR biologics OR biosimilars OR biopharmaceutical* OR ((biologic* OR biosimilar* OR immunomodulat*) NEAR/2 (factor* OR agent* OR drug* OR pharmaceutical*)) OR ((monoclonal) NEAR/1 (antibod*)) OR infliximab* OR Remicade OR ustekinumab* OR stelara OR vedolizumab* OR entyvio OR adalimumab* OR Humira OR golimumab* OR Simponi OR certolizumab* OR Cimzia)**)** **AND** **(**(((inflammatory-bowel) NEAR/2 (disease* OR disorder*)) OR Crohn* OR ileocolitis OR ((regional) NEAR/2 (enterit* OR ileiti*)) OR ((ulcer* OR mucos* OR granulomat*) NEAR/2 (colitis OR enterit*)) OR IBD)**)** **AND** ((effic* OR effectiv* OR response* OR nonresponse* OR ((treatment* OR therap* OR drug* OR pharmacolog* OR physiologic* OR clinical) NEAR/5 (success* OR effect* OR potential OR outcome* OR failure*)))) **NOT** ((animal* OR rat OR rats OR mouse OR mice) NOT (human*))) **AND** DT=Article

***Google Scholar: 200*** *(top relevant)*

Biologics|biologicals|biosimilars|"biologic|biosimilar|immunomodulating agent|agents|factors|factor" IBD|Crohn|"inflammatory * disease|disorder" efficacy|effectivity|effectiveness|response "treatment|therapy|clinical effect|outcome"

# Appendix 2: Study criteria for detailed review

On order to be considered for detailed review, studies had to fulfil the following criteria:

Types of study: All study types (e.g. observational, retrospective) were allowed

Types of participants: The aim of this study was to investigate adult patients (18 years or older) with inflammatory bowel disease . Studies were allowed to investigate both patients with a first presentation and patients with a relapse of previously established disease. At least 30 patients had to be included in the study.

Types of interventions: Any of the following drugs: infliximab, adalimumab, golimumab, certolizumab pegol, vedolizumab or ustekinumab. All durations and formulations were allowed.

Types of outcome measures: Any assessment of patient sex on the endoscopically assessed efficacy of the interventions described above. Any assessment of patient sex on the occurrence or severity adverse events. For example a regression analysis where patient sex is assessed as a factor.

# Appendix 3: Study criteria for inclusion in review

Upon detailed review, studies had to fulfill the following criteria in order to be eligible for inclusion:

1. Study design
   1. Any study, regardless of study design (e.g. retrospective or prospective, controlled or uncontrolled, blinded or unblinded)
      1. Study results that were only present in abstract form (e.g. conference abstract) were not included
2. Study population
   1. Adults (18 years or older)
   2. With inflammatory bowel disease
   3. At least 30 patients included in the study
3. Intervention and control group
   1. Any of the following drugs: adalimumab, certolizumab pegol, golimumab, infliximab, ustekinumab or vedolizumab
   2. Biological drug therapy must be prescribed in order to treat the inflammatory bowel disease
4. Outcome measure
   1. Primary: the influence of patient sex on endoscopic efficacy outcomes (e.g. endoscopic response or remission)
   2. Secondary: the influence of patient sex on adverse events (e.g. frequency or severity of adverse events)

#

# Appendix 4: References to studies excluded upon detailed review

1. Adar T, Faleck D, Sasidharan S, Cushing K, Borren NZ, Nalagatla N, et al. Comparative safety and effectiveness of tumor necrosis factor α antagonists and vedolizumab in elderly IBD patients: a multicentre study. Aliment Pharmacol Ther. 2019;49(7):873-9.

2. Adedokun OJ, Sandborn WJ, Feagan BG, Rutgeerts P, Xu Z, Marano CW, et al. Association between serum concentration of infliximab and efficacy in adult patients with ulcerative colitis. Gastroenterology. 2014;147(6):1296-307.e5.

3. Af Björkesten CG, Nieminen U, Sipponen T, Turunen U, Arkkila P, Färkkilä M. Mucosal healing at 3 months predicts long-term endoscopic remission in anti-TNF-treated luminal Crohn's disease. Scand J Gastroenterol. 2013;48(5):543-51.

4. Allez M, Vermeire S, Mozziconacci N, Michetti P, Laharie D, Louis E, et al. The efficacy and safety of a third anti-TNF monoclonal antibody in Crohn's disease after failure of two other anti-TNF antibodies. Aliment Pharmacol Ther. 2010;31(1):92-101.

5. Amiot A, Grimaud JC, Peyrin-Biroulet L, Filippi J, Pariente B, Roblin X, et al. Effectiveness and Safety of Vedolizumab Induction Therapy for Patients With Inflammatory Bowel Disease. Clin Gastroenterol Hepatol. 2016;14(11):1593-601.e2.

6. Amiot A, Serrero M, Peyrin-Biroulet L, Filippi J, Pariente B, Roblin X, et al. One-year effectiveness and safety of vedolizumab therapy for inflammatory bowel disease: a prospective multicentre cohort study. Aliment Pharmacol Ther. 2017;46(3):310-21.

7. Ampuero J, Rojas-Feria M, Castro-Fernandez M, Millan-Lorenzo M, Guerrero-Jimenez P, Romero-Gomez M. Remission maintained by monotherapy after biological plus immunosuppressive combination for Crohn's disease in clinical practice. J Gastroenterol Hepatol. 2016;31(1):112-8.

8. Appau KA, Fazio VW, Shen B, Church JM, Lashner B, Remzi F, et al. Use of infliximab within 3 months of ileocolonic resection is associated with adverse postoperative outcomes in Crohn's patients. J Gastrointest Surg. 2008;12(10):1738-44.

9. Argüelles-Arias F, Guerra Veloz MF, Perea Amarillo R, Vilches-Arenas A, Castro Laria L, Maldonado Pérez B, et al. Effectiveness and Safety of CT-P13 (Biosimilar Infliximab) in Patients with Inflammatory Bowel Disease in Real Life at 6 Months. Dig Dis Sci. 2017;62(5):1305-12.

10. Armuzzi A, Biancone L, Daperno M, Coli A, Pugliese D, Annese V, et al. Adalimumab in active ulcerative colitis: A "real-life" observational study. Dig Liver Dis. 2013;45(9):738-43.

11. Baert F, Drobne D, Gils A, Vande Casteele N, Hauenstein S, Singh S, et al. Early trough levels and antibodies to infliximab predict safety and success of reinitiation of infliximab therapy. Clin Gastroenterol Hepatol. 2014;12(9):1474-81.e2.

12. Baert F, Moortgat L, Van Assche G, Caenepeel P, Vergauwe P, De Vos M, et al. Mucosal Healing Predicts Sustained Clinical Remission in Patients With Early-Stage Crohn's Disease. Gastroenterology. 2010;138(2):463-8.

13. Bálint A, Farkas K, Palatka K, Lakner L, Miheller P, Rácz I, et al. Efficacy and safety of adalimumab in ulcerative colitis refractory to conventional therapy in routine clinical practice. J Crohn's Colitis. 2016;10(1):26-30.

14. Barber GE, Yajnik V, Khalili H, Giallourakis C, Garber J, Xavier R, et al. Genetic Markers Predict Primary Non-Response and Durable Response to Anti-TNF Biologic Therapies in Crohn's Disease. Am J Gastroenterol. 2016;111(12):1816-22.

15. Battat R, Dulai PS, Vande Casteele N, Evans E, Hester KD, Webster E, et al. Biomarkers Are Associated with Clinical and Endoscopic Outcomes with Vedolizumab Treatment in Ulcerative Colitis. Inflammatory Bowel Dis. 2019;25(2):410-20.

16. Battat R, Kopylov U, Bessissow T, Bitton A, Cohen A, Jain A, et al. Association Between Ustekinumab Trough Concentrations and Clinical, Biomarker, and Endoscopic Outcomes in Patients With Crohn's Disease. Clin Gastroenterol Hepatol. 2017;15(9):1427-34.e2.

17. Bau M, Zacharias P, Ribeiro DA, Boaron L, Steckert Filho A, Kotze PG. Safety profile of anti-TNF therapy in Crohn’s disease management: A Brazilian single-center direct retrospective comparison between infliximab and adalimumab. Arq Gastroenterol. 2017;54(4):328-32.

18. Ben-Horin S, Chowers Y, Ungar B, Kopylov U, Loebstein R, Weiss B, et al. Undetectable anti-TNF drug levels in patients with long-term remission predict successful drug withdrawal. Aliment Pharmacol Ther. 2015;42(3):356-64.

19. Ben-Horin S, Ungar B, Kopylov U, Lahat A, Yavzori M, Fudim E, et al. Safety, efficacy and pharmacokinetics of vedolizumab in patients with simultaneous exposure to an anti-tumour necrosis factor. Aliment Pharmacol Ther. 2018;47(8):1117-25.

20. Bernardo S, Fernandes SR, Gonçalves AR, Valente A, Baldaia C, Santos PM, et al. Predicting the Course of Disease in Hospitalized Patients With Acute Severe Ulcerative Colitis. Inflamm Bowel Dis. 2019;25(3):541-6.

21. Björkesten CGA, Nieminen U, Turunen U, Arkkila PE, Sipponen T, Färkkilä MA. Endoscopic monitoring of infliximab therapy in Crohn's disease. Inflammatory Bowel Dis. 2011;17(4):947-53.

22. Boktor M, Motlis A, Aravantagi A, Sheth A, Jordan P, Morris J, et al. Substitution with alternative Anti-TNFα therapy (SAVANT) - Outcomes of a Crohn's disease cohort undergoing substitution therapy with certolizumab. Inflammatory Bowel Dis. 2016;22(6):1353-61.

23. Bosca-Watts MM, Cortes X, Iborra M, Huguet JM, Sempere L, Garcia G, et al. Short-term effectiveness of golimumab for ulcerative colitis: Observational multicenter study. World J Gastroenterol. 2016;22(47):10432-9.

24. Bossuyt P, Baert F, D'Heygere F, Nakad A, Reenaers C, Fontaine F, et al. Early mucosal healing predicts favorable outcomes in patients with moderate to severe ulcerative colitis treated with golimumab: Data from the real-life BE-SMART cohort. Inflammatory Bowel Dis. 2019;25(1):156-62.

25. Bouguen G, Laharie D, Nancey S, Hebuterne X, Flourie B, Filippi J, et al. Efficacy and safety of adalimumab 80 mg weekly in luminal crohn's disease. Inflammatory Bowel Dis. 2015;21(5):1047-53.

26. Bouguen G, Levesque BG, Pola S, Evans E, Sandborn WJ. Feasibility of Endoscopic Assessment and Treating to Target to Achieve Mucosal Healing in Ulcerative Colitis. Inflamm Bowel Dis. 2014;20(2):231-9.

27. Bouguen G, Levesque BG, Pola S, Evans E, Sandborn WJ. Endoscopic Assessment and Treating to Target Increase the Likelihood of Mucosal Healing in Patients With Crohn's Disease. Clin Gastroenterol Hepatol. 2014;12(6):978-85.

28. Brandse JF, Vos LM, Jansen J, Schakel T, Ponsioen CI, van den Brink GR, et al. Serum Concentration of Anti-TNF Antibodies, Adverse Effects and Quality of Life in Patients with Inflammatory Bowel Disease in Remission on Maintenance Treatment. J Crohns Colitis. 2015;9(11):973-81.

29. Buer LCT, Moum BA, Cvancarova M, Warren DJ, Bolstad N, Medhus AW, et al. Real world data on effectiveness, safety and therapeutic drug monitoring of vedolizumab in patients with inflammatory bowel disease. A single center cohort. Scand J Gastroenterol. 2019;54(1):41-8.

30. Castiglione F, Mainenti P, Testa A, Imperatore N, De Palma GD, Maurea S, et al. Cross-sectional evaluation of transmural healing in patients with Crohn's disease on maintenance treatment with anti-TNF alpha agents. Dig Liver Dis. 2017;49(5):484-9.

31. Castiglione F, Testa A, Rea M, De Palma GD, Diaferia M, Musto D, et al. Transmural healing evaluated by bowel sonography in patients with Crohn's disease on maintenance treatment with biologics. Inflammatory Bowel Dis. 2013;19(9):1928-34.

32. Caviglia R, Ribolsi M, Rizzi M, Emerenziani S, Annunziata ML, Cicala M. Maintenance of remission with infliximab in inflammatory bowel disease: Efficacy and safety long-term follow-up. World J Gastroenterol. 2007;13(39):5238-44.

33. Chang CW, Wei SC, Chou JW, Hsu TC, Chuang CH, Lin CP, et al. Safety and Efficacy of Adalimumab for Patients With Moderate to Severe Crohn's Disease: The Taiwan Society of Inflammatory Bowel Disease (TSIBD) Study. Intestinal Res. 2014;12(4):287-92.

34. Chaparro M, Garre A, Ricart E, Iborra M, Mesonero F, Vera I, et al. Short and long-term effectiveness and safety of vedolizumab in inflammatory bowel disease: results from the ENEIDA registry. Aliment Pharmacol Ther. 2018;48(8):839-51.

35. Chaparro M, Martínez-Montiel P, Van Domselaar M, Bermejo F, Pérez-Calle JL, Casis B, et al. Intensification of infliximab therapy in Crohn's disease: Efficacy and safety. J Crohn's Colitis. 2012;6(1):62-7.

36. Chiba M, Tsuji T, Nakane K, Tsuda S, Ishii H, Ohno H, et al. Induction with Infliximab and a Plant-Based Diet as First-Line (IPF) Therapy for Crohn Disease: A Single-Group Trial. Perm j. 2017;21:17-009.

37. Choi CH, Song ID, Kim YH, Koo JS, Kim YS, Kim JS, et al. Efficacy and safety of infliximab therapy and predictors of response in korean patients with crohn’s disease: A nationwide, multicenter study. Yonsei Med J. 2016;57(6):1376-85.

38. Christensen B, Colman RJ, Micic D, Gibson PR, Goeppinger SR, Yarur A, et al. Vedolizumab as Induction and Maintenance for Inflammatory Bowel Disease: 12-month Effectiveness and Safety. Inflammatory Bowel Dis. 2018;24(4):849-60.

39. Christensen B, Gibson PR, Micic D, Colman RJ, Goeppinger SR, Kassim O, et al. Safety and Efficacy of Combination Treatment With Calcineurin Inhibitors and Vedolizumab in Patients With Refractory Inflammatory Bowel Disease. Clin Gastroenterol Hepatol. 2019;17(3):486-93.

40. Christensen B, Hanauer SB, Erlich J, Kassim O, Gibson PR, Turner JR, et al. Histologic Normalization Occurs in Ulcerative Colitis and Is Associated With Improved Clinical Outcomes. Clin Gastroenterol Hepatol. 2017;15(10):1557-+.

41. Collins M, Sarter H, Gower-Rousseau C, Koriche D, Libier L, Nachury M, et al. Previous Exposure to Multiple Anti-TNF Is Associated with Decreased Efficiency in Preventing Postoperative Crohn's Disease Recurrence. J Crohns Colitis. 2017;11(3):281-8.

42. Colombel JF, Jharap B, Sandborn WJ, Feagan B, Peyrin-Biroulet L, Eichner SF, et al. Effects of concomitant immunomodulators on the pharmacokinetics, efficacy and safety of adalimumab in patients with Crohn's disease or ulcerative colitis who had failed conventional therapy. Aliment Pharmacol Ther. 2017;45(1):50-62.

43. Colombel JF, Panaccione R, Bossuyt P, Lukas M, Baert F, Vanasek T, et al. Effect of tight control management on Crohn's disease (CALM): a multicentre, randomised, controlled phase 3 trial. Lancet. 2018;390(10114):2779-89.

44. Colombel JF, Reinisch W, Mantzaris GJ, Kornbluth A, Rutgeerts P, Tang KL, et al. Randomised clinical trial: Deep remission in biologic and immunomodulator naïve patients with Crohn's disease - A SONIC post hoc analysis. Aliment Pharmacol Ther. 2015;41(8):734-46.

45. Colombel JF, Rutgeerts PJ, Sandborn WJ, Yang M, Camez A, Pollack PF, et al. Adalimumab induces deep remission in patients with Crohn's disease. Clin Gastroenterol Hepatol. 2014;12(3):414-22.e5.

46. Colombel JF, Sandborn WJ, Allez M, Dupas J, Dewit O, D'Haens G, et al. Association between plasma concentrations of certolizumab pegol and endoscopic outcomes of patients with Crohn's disease. Clin Gastroenterol Hepatol. 2014;12(3):423-31.e1.

47. Colombel JF, Sandborn WJ, Ghosh S, Wolf DC, Panaccione R, Feagan B, et al. Four-year maintenance treatment with adalimumab in patients with moderately to severely active ulcerative Colitis: Data from ULTRA 1, 2, and 3. Am J Gastroenterol. 2014;109(11):1771-80.

48. Colombel JF, Sandborn WJ, Panaccione R, Robinson AM, Lau W, Li J, et al. Adalimumab safety in global clinical trials of patients with Crohn's disease. Inflammatory Bowel Dis. 2009;15(9):1308-19.

49. Colombel JF, Sandborn WJ, Reinisch W, Mantzaris GJ, Kornbluth A, Rachmilewitz D, et al. Infliximab, azathioprine, or combination therapy for Crohn's disease. New Engl J Med. 2010;362(15):1383-95.

50. Cotter J, Dias de Castro F, Moreira MJ, Rosa B. Tailoring Crohn's disease treatment: The impact of small bowel capsule endoscopy. J Crohn's Colitis. 2014;8(12):1610-5.

51. Dai C, Liu WX, Jiang M, Sun MJ. Mucosal healing did not predict sustained clinical remission in patients with IBD after discontinuation of one-year infliximab therapy. PLoS ONE. 2014;9(10).

52. de Barcelos IF, Kotze PG, Spinelli A, Suzuki Y, Teixeira FV, de Albuquerque IC, et al. Factors affecting the incidence of early endoscopic recurrence after ileocolonic resection for Crohn's disease: a multicentre observational study. Colorectal Dis. 2017;19(1):O39-O45.

53. De Bruyn M, Machiels K, Vandooren J, Lemmens B, Van Lommel L, Breynaert C, et al. Infliximab restores the dysfunctional matrix remodeling protein and growth factor gene expression in patients with inflammatory bowel disease. Inflammatory Bowel Dis. 2014;20(2):339-52.

54. De Cruz P, Kamm MA, Hamilton AL, Ritchie KJ, Krejany EO, Gorelik A, et al. Crohn's disease management after intestinal resection: A randomised trial. Lancet. 2015;385(9976):1406-17.

55. De Silva PSA, Nguyen DD, Sauk J, Korzenik J, Yajnik V, Ananthakrishnan AN. Long-term outcome of a third anti-TNF monoclonal antibody after the failure of two prior anti-TNFs in inflammatory bowel disease. Aliment Pharmacol Ther. 2012;36(5):459-66.

56. De Vos M, Dewit O, D'Haens G, Baert F, Fontaine F, Vermeire S, et al. Fast and sharp decrease in calprotectin predicts remission by infliximab in anti-TNF naïve patients with ulcerative colitis. J Crohn's Colitis. 2012;6(5):557-62.

57. De Vos M, Louis EJ, Jahnsen J, Vandervoort JGP, Noman M, Dewit O, et al. Consecutive fecal calprotectin measurements to predict relapse in patients with ulcerative colitis receiving infliximab maintenance therapy. Inflammatory Bowel Dis. 2013;19(10):2111-7.

58. D'Haens G, Reinisch W, Colombel JF, Panes J, Ghosh S, Prantera C, et al. Five-year Safety Data From ENCORE, a European Observational Safety Registry for Adults With Crohn's Disease Treated With Infliximab Remicade or Conventional Therapy. J Crohns Colitis. 2017;11(6):680-9.

59. D'Haens G, Van Deventer S, Van Hogezand R, Chalmers D, Kothe C, Baert F, et al. Endoscopic and histological healing with infliximab anti-tumor necrosis factor antibodies in Crohn's disease: A european multicenter trial. Gastroenterology. 1999;116(5):1029-34.

60. D'Haens G, Vermeire S, Lambrecht G, Baert F, Bossuyt P, Pariente B, et al. Increasing Infliximab Dose Based on Symptoms, Biomarkers, and Serum Drug Concentrations Does Not Increase Clinical, Endoscopic, and Corticosteroid-Free Remission in Patients With Active Luminal Crohn's Disease. Gastroenterology. 2018;154(5):1343-51.e1.

61. Di Domenicantonio R, Trotta F, Cascini S, Agabiti N, Kohn A, Gasbarrini A, et al. Population-based cohort study on comparative effectiveness and safety of biologics in inflammatory bowel disease. Clin Epidemiol. 2018;10:203-13.

62. Domènech E, Zabana Y, Mañosa M, Garcia-Planella E, Cabré E, Gassull MA. Infliximab reintroduction is not associated to a higher rate of immune-related adverse effects in patients with inflammatory bowel disease initially treated with a three-infusion induction regimen. J Clin Gastroenterol. 2010;44(1):34-7.

63. Drobne D, Kurent T, Golob S, Švegl P, Rajar P, Hanžel J, et al. Optimised infliximab monotherapy is as effective as optimised combination therapy, but is associated with higher drug consumption in inflammatory bowel disease. Aliment Pharmacol Ther. 2019;49(7):880-9.

64. Drobne D, Kurent T, Golob S, Svegl P, Rajar P, Terzic S, et al. Success and safety of high infliximab trough levels in inflammatory bowel disease. Scand J Gastroenterol. 2018;53(8):940-6.

65. Echarri A, Ollero V, Barreiro-de Acosta M, Fernández-Villaverde A, Hernández V, Lorenzo A, et al. Clinical, biological, and endoscopic responses to adalimumab in antitumor necrosis factor-naive Crohn's disease: Predictors of efficacy in clinical practice. Eur J Gastroenterol Hepatol. 2015;27(4):430-5.

66. Farkas K, Lakatos PL, Szucs M, Pallagi-Kunstár É, Bálint A, Nagy F, et al. Frequency and prognostic role of mucosal healing in patients with Crohn's disease and ulcerative colitis after one-year of biological therapy. World J Gastroenterol. 2014;20(11):2995-3001.

67. Feagan BG, Greenberg GR, Wild G, Fedorak RN, Pare P, McDonald JWD, et al. Treatment of ulcerative colitis with a humanized antibody to the alpha(4)beta(7) integrin. N Engl J Med. 2005;352(24):2499-507.

68. Feagan BG, Rutgeerts P, Sands BE, Hanauer S, Colombel JF, Sandborn WJ, et al. Vedolizumab as induction and maintenance therapy for ulcerative colitis. New Engl J Med. 2013;369(8):699-710.

69. Feng T, Chen B, Li L, Huang S, Ben-Horin S, Qiu Y, et al. Serum Interleukin 9 Levels Predict Disease Severity and the Clinical Efficacy of Infliximab in Patients with Crohn's Disease. Inflammatory Bowel Dis. 2017;23(10):1817-24.

70. Fernández-Blanco JI, Fernández-Díaz G, Cara C, Vera MI, Olivares D, Taxonera C. Adalimumab for Induction of Histological Remission in Moderately to Severely Active Ulcerative Colitis. Dig Dis Sci. 2018;63(3):731-7.

71. Ferrante M, Colombel JF, Sandborn WJ, Reinisch W, Mantzaris GJ, Kornbluth A, et al. Validation of endoscopic activity scores in patients with Crohn's disease based on a post hoc analysis of data from SONIC. Gastroenterology. 2013;145(5):978-86.e5.

72. Fukushima K, Sugita A, Futami K, Takahashi KI, Motoya S, Kimura H, et al. Postoperative therapy with infliximab for crohn’s disease: A 2-year prospective randomized multicenter study in japan. Surg Today. 2018;48(6):584-90.

73. Geboes K, Rutgeerts P, Opdenakker G, Olson A, Patel K, Wagner CL, et al. Endoscopic and histologic evidence of persistent mucosal healing and correlation with clinical improvement following sustained infliximab treatment for Crohn's disease. Curr Med Res Opin. 2005;21(11):1741-54.

74. Gecse KB, Lovász BD, Farkas K, Banai J, Bene L, Gasztonyi B, et al. Efficacy and Safety of the Biosimilar Infliximab CT-P13 Treatment in Inflammatory Bowel Diseases: A Prospective, Multicentre, Nationwide Cohort. J Crohns Colitis. 2016;10(2):133-40.

75. Godat S, Fournier N, Safroneeva E, Juillerat P, Nydegger A, Straumann A, et al. Frequency and type of drug-related side effects necessitating treatment discontinuation in the Swiss Inflammatory Bowel Disease Cohort. Eur J Gastroenterol Hepatol. 2018;30(6):612-20.

76. Goll GL, Jorgensen KK, Sexton J, Olsen IC, Bolstad N, Lorentzen M, et al. Long-term safety and efficacy of biosimilar infliximab (CT-P13) after switching from originator infliximab: results from the 26-week open label extension of a randomized Norwegian trial. Arthritis & rheumatology. 2017;69.

77. Gonczi L, Gecse KB, Vegh Z, Kurti Z, Rutka M, Farkas K, et al. Long-term Efficacy, Safety, and Immunogenicity of Biosimilar Infliximab after One Year in a Prospective Nationwide Cohort. Inflammatory Bowel Dis. 2017;23(11):1908-15.

78. Greener T, Boland K, Steinhart AH, Silverberg MS. The unfinished symphony: Golimumab therapy for anti-tumour necrosis factor refractory Crohn's disease. J Crohn's Colitis. 2018;12(4):458-64.

79. Greenup AJ, Rosenfeld G, Bressler B. Ustekinumab use in Crohn’s disease: a Canadian tertiary care centre experience. Scand J Gastroenterol. 2017;52(12):1354-9.

80. Guerra Veloz MF, Argüelles-Arias F, Laria LC, Pérez BM, Roldan AB, Amarillo RP, et al. Loss of efficacy and safety of the switch from infliximab original to infliximab biosimilar (CT-P13) in patients with inflammatory bowel disease. World J Gastroenterol. 2018;24(46):5288-96.

81. Hall BJ, Holleran GE, Smith SM, Mahmud N, McNamara DA. A prospective 12-week mucosal healing assessment of small bowel Crohn's disease as detected by capsule endoscopy. Eur J Gastroenterol Hepatol. 2014;26(11):1253-9.

82. Hamzaoglu H, Cooper J, Alsahli M, Falchuk KR, Peppercorn MA, Farrell RJ. Safety of infliximab in Crohn's disease: a large single-center experience. Inflamm Bowel Dis. 2010;16(12):2109-16.

83. Hansen RA, Gartlehner G, Powell GE, Sandler RS. Serious adverse events with infliximab: analysis of spontaneously reported adverse events. Clin Gastroenterol Hepatol. 2007;5(6):729-35.

84. Harris KA, Horst S, Gadani A, Nohl A, Annis K, Duley C, et al. Patients with Refractory Crohn's Disease Successfully Treated with Ustekinumab. Inflammatory Bowel Dis. 2016;22(2):397-401.

85. Hébuterne X, Lémann M, Bouhnik Y, Dewit O, Dupas JL, Mross M, et al. Endoscopic improvement of mucosal lesions in patients with moderate to severe ileocolonic Crohn's disease following treatment with certolizumab pegol. Gut. 2013;62(2):201-8.

86. Hendler SA, Cohen BL, Colombel JF, Sands BE, Mayer L, Agarwal S. High-dose infliximab therapy in Crohn's disease: Clinical experience, safety, and efficacy. J Crohn's Colitis. 2015;9(3):266-75.

87. Hibi T, Imai Y, Murata Y, Matsushima N, Zheng R, Gasink C. Efficacy and safety of ustekinumab in Japanese patients with moderately to severely active Crohn's disease: a subpopulation analysis of phase 3 induction and maintenance studies. Intestinal Res. 2017;15(4):475-86.

88. Hibi T, Imai Y, Senoo A, Ohta K, Ukyo Y. Efficacy and safety of golimumab 52-week maintenance therapy in Japanese patients with moderate to severely active ulcerative colitis: a phase 3, double-blind, randomized, placebo-controlled study-(PURSUIT-J study). J Gastroenterol. 2017;52(10):1101-11.

89. Hinojosa J, Gomollón F, García S, Bastida G, Cabriada JL, Saro C, et al. Efficacy and safety of short-term adalimumab treatment in patients with active Crohn's disease who lost response or showed intolerance to infliximab: A prospective, open-label, multicentre trial. Aliment Pharmacol Ther. 2007;25(4):409-18.

90. Iborra M, Pérez-Gisbert J, Bosca-Watts MM, López-García A, García-Sánchez V, López-Sanromán A, et al. Effectiveness of adalimumab for the treatment of ulcerative colitis in clinical practice: comparison between anti-tumour necrosis factor-naïve and non-naïve patients. J Gastroenterol. 2017;52(7):788-99.

91. Imaeda H, Bamba S, Takahashi K, Fujimoto T, Ban H, Tsujikawa T, et al. Relationship between serum infliximab trough levels and endoscopic activities in patients with Crohn's disease under scheduled maintenance treatment. J Gastroenterol. 2014;49(4):674-82.

92. Järnerot G, Hertervig E, Friis-Liby I, Blomquist L, Karlén P, Grännö C, et al. Infliximab as rescue therapy in severe to moderately severe ulcerative colitis: A randomized, placebo-controlled study. Gastroenterology. 2005;128(7):1805-11.

93. Jharap B, Sandborn WJ, Reinisch W, D'Haens G, Robinson AM, Wang W, et al. Randomised clinical study: Discrepancies between patient-reported outcomes and endoscopic appearance in moderate to severe ulcerative colitis. Aliment Pharmacol Ther. 2015;42(9):1082-92.

94. Jung YS, Park DI, Kim YH, Lee JH, Seo PJ, Cheon JH, et al. Efficacy and safety of CT-P13, a biosimilar of infliximab, in patients with inflammatory bowel disease: A retrospective multicenter study. J Gastroenterol Hepatol. 2015;30(12):1705-12.

95. Jürgens M, Mahachie John JM, Cleynen I, Schnitzler F, Fidder H, van Moerkercke W, et al. Levels of C-reactive Protein Are Associated With Response to Infliximab Therapy in Patients With Crohn's Disease. Clin Gastroenterol Hepatol. 2011;9(5):421-7.e1.

96. Kamat N, Kedia S, Ghoshal UC, Nehra A, Makharia G, Sood A, et al. Effectiveness and safety of adalimumab biosimilar in inflammatory bowel disease: A multicenter study. Indian J Gastroenterol. 2019.

97. Kaniewska M, Moniuszko A, Rydzewska G. The efficacy and safety of the biosimilar product (Inflectra®) compared to the reference drug (Remicade®) in rescue therapy in adult patients with ulcerative colitis. Prz Gastroenterol. 2017;12(3):169-74.

98. Kim NH, Lee JH, Hong SN, Yoon H, Kang HW, Lee SH, et al. Long-term efficacy and safety of CT-P13, a biosimilar of infliximab, in patients with inflammatory bowel disease: A retrospective multicenter study. J Gastroenterol Hepatol. 2019.

99. Kiss LS, Lovasz BD, Golovics PA, Vegh Z, Farkas K, Molnar T, et al. Levels of anti-double-strained DNA but not antinuclear antibodies are associated with treatment efficacy and adverse outcomes in Crohn's disease patients treated with anti-TNFα. J Gastrointest Liver Dis. 2013;22(2):135-40.

100. Kolar M, Duricova D, Bortlik M, Hruba V, Machkova N, Mitrova K, et al. Biosimilar infliximab in anti-TNF-naïve IBD patients - 1-year clinical follow-up. Gastroenterol Hepatol. 2016;70(6):514-22.

101. Kolar M, Duricova D, Bortlik M, Hruba V, MacHkova N, Mitrova K, et al. Infliximab Biosimilar (Remsima™) in Therapy of Inflammatory Bowel Diseases Patients: Experience from One Tertiary Inflammatory Bowel Diseases Centre. Dig Dis. 2017;35(1-2):91-100.

102. Kopylov U, Avni-Biron I, Ron Y, Koslowsky B, Waterman M, Daher S, et al. Effectiveness and safety of vedolizumab for maintenance treatment in inflammatory bowel disease—The Israeli real world experience. Dig Liver Dis. 2019;51(1):68-74.

103. Kopylov U, Ron Y, Avni-Biron I, Koslowsky B, Waterman M, Daher S, et al. Efficacy and Safety of Vedolizumab for Induction of Remission in Inflammatory Bowel Disease-the Israeli Real-World Experience. Inflammatory Bowel Dis. 2017;23(3):404-8.

104. Kopylov U, Verstockt B, Biedermann L, Sebastian S, Pugliese D, Sonnenberg E, et al. Effectiveness and safety of vedolizumab in anti-TNF-naïve patients with inflammatory bowel disease-a multicenter retrospective European study. Inflammatory Bowel Dis. 2018;24(11):2442-51.

105. Kotze PG, Spinelli A, da Silva RN, de Barcelos IF, Teixeira FV, Saad-Hossne R, et al. Conventional Versus Biological Therapy for Prevention of Postoperative Endoscopic Recurrence in Patients With Crohn's Disease: an International, Multicenter, and Observational Study. Intestinal Res. 2015;13(3):259-65.

106. Kotze PG, Yamamoto T, Danese S, Suzuki Y, Teixeira FV, De Albuquerque IC, et al. Direct retrospective comparison of adalimumab and infliximab in preventing early postoperative endoscopic recurrence after ileocaecal resection for Crohn's disease: Results from the MULTIPER database. J Crohn's Colitis. 2015;9(7):541-7.

107. Kulaylat AS, Kulaylat AN, Schaefer EW, Tinsley A, Williams E, Koltun W, et al. Association of preoperative anti-tumor necrosis factor therapy with adverse postoperative outcomes in patients undergoing abdominal surgery for ulcerative colitis. JAMA Surg. 2017;152(8):e171538.

108. Kurnool S, Nguyen NH, Proudfoot J, Dulai PS, Boland BS, Vande Casteele N, et al. High body mass index is associated with increased risk of treatment failure and surgery in biologic-treated patients with ulcerative colitis. Aliment Pharmacol Ther. 2018;47(11):1472-9.

109. Laharie D, Filippi J, Roblin X, Nancey S, Chevaux JB, Hébuterne X, et al. Impact of mucosal healing on long-term outcomes in ulcerative colitis treated with infliximab: A multicenter experience. Aliment Pharmacol Ther. 2013;37(10):998-1004.

110. Lee KM, Jeen YT, Cho JY, Lee CK, Koo JS, Park DI, et al. Efficacy, safety, and predictors of response to infliximab therapy for ulcerative colitis: A Korean multicenter retrospective study. J Gastroenterol Hepatol. 2013;28(12):1829-33.

111. Lees CW, Ali AI, Thompson AI, Ho GT, Forsythe RO, Marquez L, et al. The safety profile of anti-tumour necrosis factor therapy in inflammatory bowel disease in clinical practice: analysis of 620 patient-years follow-up. Aliment Pharmacol Ther. 2009;29(3):286-97.

112. Lees CW, Heys D, Ho GT, Noble CL, Shand AG, Mowat C, et al. A retrospective analysis of the efficacy and safety of infliximab as rescue therapy in acute severe ulcerative colitis. Aliment Pharmacol Ther. 2007;26(3):411-9.

113. Lehtola E, Haapamäki J, Färkkilä MA. Outcome of inflammatory bowel disease patients treated with TNF-α inhibitors: two-year follow-up. Scand J Gastroenterol. 2016;51(12):1476-81.

114. LÉmann M, Mary J, Duclos B, Veyrac M, Dupas J, Delchier JC, et al. Infliximab Plus Azathioprine for Steroid-Dependent Crohn's Disease Patients: A Randomized Placebo-Controlled Trial. Gastroenterology. 2006;130(4):1054-61.

115. Lenti MV, Levison S, Eliadou E, Willert R, Kemp K, Carter A, et al. A real-world, long-term experience on effectiveness and safety of vedolizumab in adult patients with inflammatory bowel disease: The Cross Pennine study. Dig Liver Dis. 2018;50(12):1299-304.

116. Leung CM, Tang W, Kyaw M, Niamul G, Aniwan S, Limsrivilai J, et al. Endoscopic and Histological Mucosal Healing in Ulcerative Colitis in the First Year of Diagnosis: Results from a Population-based Inception Cohort from Six Countries in Asia. Journal of Crohns & Colitis. 2017;11(12):1440-8.

117. Li Y, Lopez R, Queener E, Shen B. Adalimumab therapy in Crohn's disease of the ileal pouch. Inflammatory Bowel Dis. 2012;18(12):2232-9.

118. Lichtiger S, Binion DG, Wolf DC, Present DH, Bensimon AG, Wu E, et al. The CHOICE trial: Adalimumab demonstrates safety, fistula healing, improved quality of life and increased work productivity in patients with Crohns disease who failed prior infliximab therapy. Aliment Pharmacol Ther. 2010;32(10):1228-39.

119. Liefferinckx C, Minsart C, Cremer A, Amininejad L, Tafciu V, Quertinmont E, et al. Early vedolizumab trough levels at induction in inflammatory bowel disease patients with treatment failure during maintenance. Eur J Gastroenterol Hepatol. 2019;31(4):478-85.

120. Liu J, Sylwestrzak G, Ruggieri AP, DeVries A. Intravenous versus subcutaneous anti-TNF-alpha agents for Crohn's disease: A comparison of effectiveness and safety. J Managed Care Pharm. 2015;21(7):559-66.

121. Lobaton T, Ferrante M, Rutgeerts P, Ballet V, Van Assche G, Vermeire S. Efficacy and safety of anti-TNF therapy in elderly patients with inflammatory bowel disease. Aliment Pharmacol Ther. 2015;42(4):441-51.

122. Loftus EV, Jr., Colombel JF, Schreiber S, Randall CW, Regueiro M, Ali T, et al. Safety of Long-term Treatment With Certolizumab Pegol in Patients With Crohn's Disease, Based on a Pooled Analysis of Data From Clinical Trials. Clin Gastroenterol Hepatol. 2016;14(12):1753-62.

123. Lopez-San Roman A, Vera-Mendoza I, Domènech E, Taxonera C, Ruiz VV, Marín-Jiménez I, et al. Adalimumab vs azathioprine in the prevention of postoperative Crohn's disease recurrence. A GETECCU randomised trial. J Crohn's Colitis. 2017;11(11):1293-301.

124. Lykowska-Szuber L, Klimczak K, Eder P, Krela-Kazmierczak I, Stawczyk-Eder K, Michalak M, et al. Diagnostic importance of faecal markers in long-term monitoring of anti-TNF-alpha therapy in primary responders with Crohn's disease. Gastroenterology Review-Przeglad Gastroenterologiczny. 2016;11(4):232-8.

125. Ma C, Fedorak RN, Kaplan GG, Dieleman LA, Devlin SM, Stern N, et al. Clinical, endoscopic and radiographic outcomes with ustekinumab in medically-refractory Crohn's disease: real world experience from a multicentre cohort. Aliment Pharmacol Ther. 2017;45(9):1232-43.

126. Ma C, Fedorak RN, Kaplan GG, Dieleman LA, Devlin SM, Stern N, et al. Long-term Maintenance of Clinical, Endoscopic, and Radiographic Response to Ustekinumab in Moderate-to-Severe Crohn's Disease: Real-world Experience from a Multicenter Cohort Study. Inflammatory Bowel Dis. 2017;23(5):833-9.

127. Magro F, Afonso J, Lopes S, Coelho R, Gonçalves R, Caldeira P, et al. Calprotectin and the Magnitude of Antibodies to Infliximab in Clinically-stable Ulcerative Colitis Patients are More Relevant Than Infliximab Trough Levels and Pharmacokinetics for Therapeutic Escalation. EBioMedicine. 2017;21:123-30.

128. Mao R, Qiu Y, Chen BL, Zhang SH, Feng R, He Y, et al. Factors associated with the achievement of mucosal healing in Crohn's disease: the benefit of endoscopic monitoring in treating to target. Therapeutic Advances in Gastroenterology. 2017;10(6):453-63.

129. Marehbian J, Arrighi HM, Hass S, Tian HJ, Sandborn WJ. Adverse Events Associated With Common Therapy Regimens for Moderate-to-Severe Crohn's Disease. Am J Gastroenterol. 2009;104(10):2524-33.

130. Martineau C, Flourié B, Wils P, Vaysse T, Altwegg R, Buisson A, et al. Efficacy and safety of golimumab in Crohn's disease: a French national retrospective study. Aliment Pharmacol Ther. 2017;46(11-12):1077-84.

131. Matsumoto T, Motoya S, Watanabe K, Hisamatsu T, Nakase H, Yoshimura N, et al. Adalimumab monotherapy and a combination with azathioprine for Crohn's disease: A prospective, randomized trial. J Crohn's Colitis. 2016;10(11):1259-66.

132. Meyer A, Rudant J, Drouin J, Weill A, Carbonnel F, Coste J. Effectiveness and safety of reference infliximab and biosimilar in Crohn disease: A French equivalence study. Ann Intern Med. 2019;170(2):99-107.

133. Miheller P, Lakatos PL, Horváth G, Molnár T, Szamosi T, Czeglédi Z, et al. Efficacy and safety of infliximab induction therapy in Crohn's Disease in Central Europe - A Hungarian nationwide observational study. BMC Gastroenterol. 2009;9:66.

134. Molander P, Sipponen T, Kemppainen H, Jussila A, Blomster T, Koskela R, et al. Achievement of deep remission during scheduled maintenance therapy with TNFalpha-blocking agents in IBD. J Crohns Colitis. 2013;7(9):730-5.

135. Moon W, Pestana L, Becker B, Loftus EV, Hanson KA, Bruining DH, et al. Efficacy and safety of certolizumab pegol for Crohn's disease in clinical practice. Aliment Pharmacol Ther. 2015;42(4):428-40.

136. Mortensen C, Caspersen S, Christensen NL, Svenningsen L, Thorsgaard N, Christensen LA, et al. Treatment of acute ulcerative colitis with infliximab, a retrospective study from three Danish hospitals. J Crohn's Colitis. 2011;5(1):28-33.

137. Motoya S, Watanabe K, Ogata H, Kanai T, Matsui T, Suzuki Y, et al. Vedolizumab in Japanese patients with ulcerative colitis: A Phase 3, randomized, double-blind, placebo-controlled study. PLoS ONE. 2019;14(2).

138. Muñoz-Villafranca C, Ortiz de Zarate J, Arreba P, Higuera R, Gómez L, Ibáñez S, et al. Adalimumab treatment of anti-TNF-naïve patients with ulcerative colitis: Deep remission and response factors. Dig Liver Dis. 2018;50(8):812-9.

139. Nakarai A, Kato J, Hiraoka S, Inokuchi T, Takei D, Moritou Y, et al. Prognosis of ulcerative colitis differs between patients with complete and partial mucosal healing, which can be predicted from the platelet count. World J Gastroenterol. 2014;20(48):18367-74.

140. Narula N, Kainz S, Petritsch W, Haas T, Feichtenschlager T, Novacek G, et al. The efficacy and safety of either infliximab or adalimumab in 362 patients with anti-TNF-α naïve Crohn's disease. Aliment Pharmacol Ther. 2016;44(2):170-80.

141. Noman M, Ferrante M, Bisschops R, De Hertogh G, Van den Broeck K, Rans K, et al. Vedolizumab induces long-term mucosal healing in patients with Crohn's disease and ulcerative colitis. J Crohn's Colitis. 2017;11(9):1085-9.

142. Nuki Y, Esaki M, Asano K, Maehata Y, Umeno J, Moriyama T, et al. Comparison of the therapeutic efficacy and safety between tacrolimus and infliximab for moderate-to-severe ulcerative colitis: A single center experience. Scand J Gastroenterol. 2016;51(6):700-5.

143. O'Connell J, Rowan C, Stack R, Harkin G, Parihar V, Chan G, et al. Golimumab effectiveness and safety in clinical practice for moderately active ulcerative colitis. Eur J Gastroenterol Hepatol. 2018;30(9):1019-26.

144. O'Donnell S, Murphy S, Anwar MM, O'Sullivan M, Breslin N, O'Connor HJ, et al. Safety of infliximab in 10 years of clinical practice. Eur J Gastroenterol Hepatol. 2011;23(7):603-6.

145. Ogata H, Watanabe M, Matsui T, Hase H, Okayasu M, Tsuchiya T, et al. Safety of adalimumab and predictors of adverse events in 1693 Japanese patients with Crohn's disease. J Crohn's Colitis. 2016;10(9):1033-41.

146. Ordas I, Rimola J, Rodriguez S, Paredes JM, Martinez-Perez MJ, Blanc E, et al. Accuracy of Magnetic Resonance Enterography in Assessing Response to Therapy and Mucosal Healing in Patients With Crohn's Disease. Gastroenterology. 2014;146(2):374-+.

147. Orlandini B, Dragoni G, Variola A, Massella A, Bagnoli S, Campi R, et al. Clinical efficacy and safety of golimumab in biologically experienced and naive patients with active ulcerative colitis: A real-life experience from two Italian IBD centers. J Dig Dis. 2018;19(8):468-74.

148. Osterman MT, Haynes K, Delzell E, Zhang J, Bewtra M, Brensinger CM, et al. Effectiveness and safety of immunomodulators with anti-tumor necrosis factor therapy in crohn's disease. Clin Gastroenterol Hepatol. 2015;13(7):1293-301.

149. Panaccione R, Ghosh S, Middleton S, Márquez JR, Scott BB, Flint L, et al. Combination therapy with infliximab and azathioprine is superior to monotherapy with either agent in ulcerative colitis. Gastroenterology. 2014;146(2):392-400.e3.

150. Panaccione R, Loftus Jr EV, Binion D, McHugh K, Alam S, Chen N, et al. Efficacy and safety of adalimumab in Canadian patients with moderate to severe Crohn's disease: Results of the adalimumab in Canadian subjects with moderate to severe Crohn's diseaSe (ACCESS) trial. Can J Gastroenterol. 2011;25(8):419-25.

151. Pellet G, Stefanescu C, Carbonnel F, Peyrin-Biroulet L, Roblin X, Allimant C, et al. Efficacy and Safety of Induction Therapy With Calcineurin Inhibitors in Combination With Vedolizumab in Patients With Refractory Ulcerative Colitis. Clin Gastroenterol Hepatol. 2019;17(3):494-501.

152. Peyrin-Biroulet L, Reinisch W, Colombel JF, Mantzaris GJ, Kornbluth A, Diamond R, et al. Clinical disease activity, C-reactive protein normalisation and mucosal healing in Crohn's disease in the SONIC trial. Gut. 2014;63(1):88-95.

153. Pouillon L, Ferrante M, Van Assche G, Rutgeerts P, Noman M, Sabino J, et al. Mucosal Healing and Long-term Outcomes of Patients With Inflammatory Bowel Diseases Receiving Clinic-Based vs Trough Concentration-Based Dosing of Infliximab. Clin Gastroenterol Hepatol. 2018;16(8):1276-83.e1.

154. Raimundo Fernandes S, Santos PM, Moura CM, Marques Da Costa P, Carvalho JR, Valente AI, et al. The use of a segmental endoscopic score may improve the prediction of clinical outcomes in acute severe ulcerative colitis. Rev Esp Enferm Dig. 2016;108(11):697-702.

155. Regueiro M, Feagan BG, Zou B, Johanns J, Blank MA, Chevrier M, et al. Infliximab Reduces Endoscopic, but Not Clinical, Recurrence of Crohn's Disease after Ileocolonic Resection. Gastroenterology. 2016;150(7):1568-78.

156. Reinisch W, Bressler B, Curtis R, Parikh A, Yang H, Rosario M, et al. Fecal calprotectin responses following induction therapy with vedolizumab in moderate to severe ulcerative colitis: A post hoc analysis of gemini 1. Inflammatory Bowel Dis. 2019;25(4):803-10.

157. Reinisch W, Colombel JF, D'Haens G, Sandborn WJ, Rutgeerts P, Geboes K, et al. Characterisation of Mucosal Healing with Adalimumab Treatment in Patients with Moderately to Severely Active Crohn's Disease: Results from the EXTEND Trial. J Crohns Colitis. 2017;11(4):425-34.

158. Reinisch W, Colombel JF, Sandborn WJ, Mantzaris GJ, Kornbluth A, Adedokun OJ, et al. Factors associated with short- and long-term outcomes oftherapy for crohn's disease. Clin Gastroenterol Hepatol. 2015;13(3):539-47.

159. Reinisch W, Sandborn WJ, Panaccione R, Huang B, Pollack PF, Lazar A, et al. 52-week efficacy of adalimumab in patients with moderately to severely active ulcerative colitis who failed corticosteroids and/or immunosuppressants. Inflammatory Bowel Dis. 2013;19(8):1700-9.

160. Robbins L, Zaghiyan K, Melmed G, Vasiliauskas E, Ahmed S, McGovern D, et al. Outcomes with Anti-Tumour Necrosis Factor-Alpha Therapy and Serology in Patients with Denovo Crohn's Disease After Ileal Pouch Anal Anastomosis. Journal of Crohns & Colitis. 2017;11(1):77-83.

161. Rutgeerts P, D'Haens G, Targan S, Vasiliauskas E, Hanauer SB, Present DH, et al. Efficacy and safety of retreatment with anti-tumor necrosis factor antibody (infliximab) to maintain remission in Crohn's disease. Gastroenterology. 1999;117(4):761-9.

162. Rutgeerts P, Diamond RH, Bala M, Olson A, Lichtenstein GR, Bao W, et al. Scheduled maintenance treatment with infliximab is superior to episodic treatment for the healing of mucosal ulceration associated with Crohn's disease. Gastrointest Endosc. 2006;63(3):433-42.

163. Rutgeerts P, Feagan BG, Lichtenstein GR, Mayer LF, Schreiber S, Colombel JF, et al. Comparison of Scheduled and Episodic Treatment Strategies of Infliximab in Crohn's Disease. Gastroenterology. 2004;126(2):402-13.

164. Rutgeerts P, Gasink C, Chan D, Lang Y, Pollack P, Colombel JF, et al. Efficacy of Ustekinumab for Inducing Endoscopic Healing in Patients With Crohn's Disease. Gastroenterology. 2018;155(4):1045-58.

165. Rutgeerts P, Van Assche G, Sandborn WJ, Wolf DC, Geboes K, Colombel JF, et al. Adalimumab induces and maintains mucosal healing in patients with Crohn's Disease: Data from the EXTEND trial. Gastroenterology. 2012;142(5):1102-11.e2.

166. Saigusa K, Matsuoka K, Sugimoto S, Arai M, Kiyohara H, Takeshita K, et al. Ulcerative colitis endoscopic index of severity is associated with long-term prognosis in ulcerative colitis patients treated with infliximab. Dig Endosc. 2016;28(6):665-70.

167. Saito E, Nagahori M, Fujii T, Ohtsuka K, Watanabe M. Efficacy of salvage therapy and its effect on operative outcomes in patients with ulcerative colitis. Digestion. 2014;89(1):55-60.

168. Samaan MA, Pavlidis P, Digby-Bell J, Johnston EL, Dhillon A, Paramsothy R, et al. Golimumab: Early experience and medium-term outcomes from two UK tertiary IBD centres. Frontline Gastroenterol. 2018;9(3):221-31.

169. Sandborn WJ, Colombel JF, D'Haens G, Van Assche G, Wolf D, Kron M, et al. One-year maintenance outcomes among patients with moderately-to-severely active ulcerative colitis who responded to induction therapy with adalimumab: Subgroup analyses from ULTRA 2. Aliment Pharmacol Ther. 2013;37(2):204-13.

170. Sandborn WJ, Colombel JF, Panaccione R, Dulai PS, Rosario M, Cao C, et al. Deep Remission with Vedolizumab in Patients with Moderately to Severely Active Ulcerative Colitis: A GEMINI 1 post hoc Analysis. J Crohn's Colitis. 2019;13(2):172-81.

171. Sandborn WJ, Feagan BG, Marano C, Zhang H, Strauss R, Johanns J, et al. Subcutaneous golimumab maintains clinical response in patients with moderate-to-severe ulcerative colitis. Gastroenterology. 2014;146(1):96-109.e1.

172. Sandborn WJ, Gasink C, Gao LL, Blank MA, Johanns J, Guzzo C, et al. Ustekinumab induction and maintenance therapy in refractory Crohn's disease. N Engl J Med. 2012;367(16):1519-28.

173. Sandborn WJ, Lee SD, Randall C, Gutierrez A, Schwartz DA, Ambarkhane S, et al. Long-term safety and efficacy of certolizumab pegol in the treatment of Crohn's disease: 7-year results from the PRECiSE 3 study. Aliment Pharmacol Ther. 2014;40(8):903-16.

174. Sandborn WJ, Rutgeerts P, Gasink C, Jacobstein D, Zou B, Johanns J, et al. Long-term efficacy and safety of ustekinumab for Crohn's disease through the second year of therapy. Aliment Pharmacol Ther. 2018;48(1):65-77.

175. Sartini A, Scaioli E, Liverani E, Bellanova M, Ricciardiello L, Bazzoli F, et al. Retention Rate, Persistence and Safety of Adalimumab in Inflammatory Bowel Disease: A Real-Life, 9-Year, Single-Center Experience in Italy. Dig Dis Sci. 2019;64(3):863-74.

176. Savarino E, Bodini G, Dulbecco P, Assandri L, Bruzzone L, Mazza F, et al. Adalimumab is more effective than azathioprine and mesalamine at preventing postoperative recurrence of Crohn's disease: A randomized controlled trial. Am J Gastroenterol. 2013;108(11):1731-42.

177. Schnitzler F, Fidder H, Ferrante M, Noman M, Arijs I, Van Assche G, et al. Mucosal healing predicts long-term outcome of maintenance therapy with infliximab in Crohn's disease. Inflammatory Bowel Dis. 2009;15(9):1295-301.

178. Schoepfer AM, Vavricka SR, Binek J, Felley C, Geyer M, Manz M, et al. Efficacy and safety of certolizumab pegol induction therapy in an unselected Crohn's disease population: Results of the FACTS survey. Inflammatory Bowel Dis. 2010;16(6):933-8.

179. Seah D, Choy MC, Gorelik A, Connell WR, Sparrow MP, Van Langenberg D, et al. Examining maintenance care following infliximab salvage therapy for acute severe ulcerative colitis. J Gastroenterol Hepatol. 2018;33(1):226-31.

180. Seo HI, Park DI, Kim TO, Kim YS, Lee SH, Kim JW, et al. The effect of infliximab on patients with ulcerative colitis in Korea. Intestinal Res. 2014;12(3):214-20.

181. Seow CH, Newman A, Irwin SP, Steinhart AH, Silverberg MS, Greenberg GR. Trough serum infliximab: A predictive factor of clinical outcome for infliximab treatment in acute ulcerative colitis. Gut. 2010;59(1):49-54.

182. Shmidt E, Kochhar G, Hartke J, Chilukuri P, Meserve J, Chaudrey K, et al. Predictors and management of loss of response to vedolizumab in inflammatory bowel disease. Inflammatory Bowel Dis. 2018;24(11):2461-7.

183. Singh S, Heien HC, Sangaralingham LR, Schilz SR, Kappelman MD, Shah ND, et al. Comparative Effectiveness and Safety of Anti–Tumor Necrosis Factor Agents in Biologic-Naive Patients With Crohn's Disease. Clin Gastroenterol Hepatol. 2016;14(8):1120-9.e6.

184. Singh S, Heien HC, Sangaralingham LR, Schilz SR, Kappelman MD, Shah ND, et al. Comparative effectiveness and safety of infliximab and adalimumab in patients with ulcerative colitis. Aliment Pharmacol Ther. 2016;43(9):994-1003.

185. Singh S, Proudfoot J, Xu R, Sandborn WJ. Obesity and Response to Infliximab in Patients with Inflammatory Bowel Diseases: Pooled Analysis of Individual Participant Data from Clinical Trials. Am J Gastroenterol. 2018;113(6):883-9.

186. Sorrentino D, Terrosu G, Paviotti A, Geraci M, Avellini C, Zoli G, et al. Early diagnosis and treatment of postoperative endoscopic recurrence of Crohn's disease: Partial benefit by infiximab - A pilot study. Dig Dis Sci. 2012;57(5):1341-8.

187. Subramaniam K, Richardson A, Dodd J, Platten J, Shadbolt B, Pavli P. Early predictors of colectomy and long-term maintenance of remission in ulcerative colitis patients treated using anti-tumour necrosis factor therapy. Intern Med J. 2014;44(5):464-70.

188. Suzuki Y, Motoya S, Hanai H, Hibi T, Nakamura S, Lazar A, et al. Four-year maintenance treatment with adalimumab in Japanese patients with moderately to severely active ulcerative colitis. J Gastroenterol. 2017;52(9):1-10.

189. Suzuki Y, Motoya S, Hanai H, Matsumoto T, Hibi T, Robinson AM, et al. Efficacy and safety of adalimumab in Japanese patients with moderately to severely active ulcerative colitis. J Gastroenterol. 2014;49(2):283-94.

190. Swoger JM, Loftus Jr EV, Tremaine WJ, Faubion WA, Pardi DS, Kane SV, et al. Adalimumab for Crohns disease in clinical practice at Mayo clinic: The first 118 patients. Inflammatory Bowel Dis. 2010;16(11):1912-21.

191. Taxonera C, López-Sanromán A, Vera-Mendoza I, Domènech E, Ruiz VV, Marín-Jiménez I, et al. Quality of life during one year of postoperative prophylactic drug therapy after intestinal resection in Crohn's patients: Results of the APPRECIA trial. Dig Liver Dis. 2019.

192. Telesco SE, Brodmerkel C, Zhang H, Kim LLL, Johanns J, Mazumder A, et al. Gene Expression Signature for Prediction of Golimumab Response in a Phase 2a Open-Label Trial of Patients With Ulcerative Colitis. Gastroenterology. 2018;155(4):1008-11.e8.

193. Tomecki R, Bartnik W, Butruk E, Kościuczyk A, Kozłowska A, Marlicz K, et al. Assessment of the efficacy, tolerance and safety of infliximab (Remicade) in the treatment of Crohn's disease - Results of an open, multicentre clinical trial. Gastroenterol Pol. 2004;11(6):537-42.

194. Tursi A, Allegretta L, Buccianti N, Valle ND, Elisei W, Forti G, et al. Effectiveness and safety of golimumab in treating outpatient ulcerative colitis: A real-life prospective, multicentre, observational study in primary inflammatory bowel diseases centers. J Gastrointest Liver Dis. 2017;26(3):239-44.

195. Tursi A, Elisei W, Faggiani R, Allegretta L, Valle ND, Forti G, et al. Effectiveness and safety of adalimumab to treat outpatient ulcerative colitis: A real-life multicenter, observational study in primary inflammatory bowel disease centers. Medicine (Baltimore). 2018;97(34):e11897.

196. Tursi A, Elisei W, Giorgetti GM, Penna A, Picchio M, Brandimarte G. Factors influencing mucosal healing in crohn's disease during infliximab treatment. Hepato-Gastroenterology. 2013;60(125):1041-6.

197. Tursi A, Elisei W, Picchio M, Penna A, Lecca PG, Forti G, et al. Managing ambulatory ulcerative colitis patients with infliximab: A long-term follow-up study in primary gastroenterology centers. Eur J Intern Med. 2014;25(8):757-61.

198. Tursi A, Elisei W, Picchio M, Penna A, Lecca PG, Forti G, et al. Effectiveness and safety of infliximab and adalimumab for ambulatory Crohn's disease patients in primary gastroenterology centres. Eur J Intern Med. 2014;25(5):485-90.

199. Vadan R, Gheorghe L, Gheorghe C, Badea M, Cotruta B, Angelescu C, et al. The long term efficacy of Infliximab therapy in Crohn's disease: The experience of a tertiary referral center from Romania. Arch Balk Med Union. 2007;42(1):5-10.

200. Van Assche G, Magdelaine-Beuzelin C, D'Haens G, Baert F, Noman M, Vermeire S, et al. Withdrawal of Immunosuppression in Crohn's Disease Treated With Scheduled Infliximab Maintenance: A Randomized Trial. Gastroenterology. 2008;134(7):1861-8.

201. Varvarynets AV, Chopei IV, Chubirko KI. Clinical and endoscopic efficacy of vedolizumab in patients with ulcerative colitis. Wiad Lek. 2018;71(2):346-9.

202. Vavricka SR, Schoepfer AM, Bansky G, Binek J, Felley C, Geyer M, et al. Efficacy and safety of certolizumab pegol in an unselected crohn's disease population: 26-week data of the FACTS II survey. Inflammatory Bowel Dis. 2011;17(7):1530-9.

203. Vavricka SR, Spasojevic M, Rogler G, Schoepfer AM, Seibold F, Borovicka J, et al. Long-Term Efficacy and Safety of Certolizumab Pegol in an Unselected Crohn's Disease Population: The FACTS III Survey. Dig Dis. 2017;35(5):423-32.

204. Verstockt B, Verstockt S, Dehairs J, Ballet V, Blevi H, Wollants WJ, et al. Low TREM1 expression in whole blood predicts anti-TNF response in inflammatory bowel disease. EBioMedicine. 2019;40:733-42.

205. Vivio EE, Kanuri N, Gilbertsen JJ, Monroe K, Dey N, Chen CH, et al. Vedolizumab effectiveness and safety over the first year of use in an IBD clinical practice. J Crohn's Colitis. 2016;10(4):402-9.

206. Watanabe M, Hibi T, Mostafa NM, Chao J, Arora V, Camez A, et al. Long-term safety and efficacy of adalimumab in Japanese patients with moderate to severe Crohn's disease. J Crohn's Colitis. 2014;8(11):1407-16.

207. Weaver KN, Gregory M, Syal G, Hoversten P, Hicks SB, Patel D, et al. Ustekinumab is effective for the treatment of crohn's disease of the pouch in a multicenter cohort. Inflammatory Bowel Dis. 2019;25(4):767-74.

208. Wils P, Bouhnik Y, Michetti P, Flourie B, Brixi H, Bourrier A, et al. Long-term efficacy and safety of ustekinumab in 122 refractory Crohn's disease patients: a multicentre experience. Aliment Pharmacol Ther. 2018;47(5):588-95.

209. Wolf D, D'Haens G, Sandborn WJ, Colombel JF, Van Assche G, Robinson AM, et al. Escalation to weekly dosing recaptures response in adalimumab-treated patients with moderately to severely active ulcerative colitis. Aliment Pharmacol Ther. 2014;40(5):486-97.

210. Wright EK, Kamm MA, De Cruz P, Hamilton AL, Selvaraj F, Princen F, et al. Anti-TNF Therapeutic Drug Monitoring in Postoperative Crohn's Disease. J Crohns Colitis. 2018;12(6):653-61.

211. Wu XL, Chen RP, Tao LP, Wu JS, Chen XR, Chen WC. Infliximab Combined with Enteral Nutrition for Managing Crohn's Disease Complicated with Intestinal Fistulas. Gastroenterol Res Pract. 2016;2016.

212. Yajnik V, Khan N, Dubinsky M, Axler J, James A, Abhyankar B, et al. Efficacy and Safety of Vedolizumab in Ulcerative Colitis and Crohn’s Disease Patients Stratified by Age. Adv Ther. 2017;34(2):542-59.

213. Yamada A, Komaki Y, Patel N, Komaki F, Pekow J, Dalal S, et al. The use of vedolizumab in preventing postoperative recurrence of Crohn's disease. Inflammatory Bowel Dis. 2018;24(3):502-9.

214. Yarur AJ, Bruss A, Naik S, Beniwal-Patel P, Fox C, Jain A, et al. Vedolizumab Concentrations Are Associated with Long-Term Endoscopic Remission in Patients with Inflammatory Bowel Diseases. Dig Dis Sci. 2019.

215. Yarur AJ, Jain A, Hauenstein SI, Quintero MA, Barkin JS, Deshpande AR, et al. Higher Adalimumab Levels Are Associated with Histologic and Endoscopic Remission in Patients with Crohn's Disease and Ulcerative Colitis. Inflammatory Bowel Dis. 2016;22(2):409-15.

216. Yarur AJ, Jain A, Sussman DA, Barkin JS, Quintero MA, Princen F, et al. The association of tissue anti-TNF drug levels with serological and endoscopic disease activity in inflammatory bowel disease: The ATLAS study. Gut. 2016;65(2):249-55.

217. Ylisaukko-oja T, Aaltonen J, Nuutinen H, Blomster T, Jussila A, Pajala M, et al. High treatment persistence rate and significant endoscopic healing among real-life patients treated with vedolizumab–a Finnish Nationwide Inflammatory Bowel Disease Cohort Study (FINVEDO)*. Scand J Gastroenterol. 2018;53(2):158-67.

218. Yoshida K, Fukunaga K, Ikeuchi H, Kamikozuru K, Hida N, Ohda Y, et al. Scheduled infliximab monotherapy to prevent recurrence of Crohn's disease following ileocolic or ileal resection: A 3-year prospective randomized open trial. Inflammatory Bowel Dis. 2012;18(9):1617-23.

219. Yu Q, Wang L, Zhang S, Feng T, Li L, Chen B, et al. The role of ABO blood groups in Crohn's disease and in monitoring response to infliximab treatment. Blood Transfusion. 2016;14(5):460-4.

220. Zabana Y, Domènech E, Mañosa M, Garcia-Planella E, Bernal I, Cabré E, et al. Infliximab safety profile and long-term applicability in inflammatory bowel disease: 9-year experience in clinical practice. Aliment Pharmacol Ther. 2010;31(5):553-60.

221. Zhang T, Wang Z, Fan R, Zhang M, Lin Y, Hong L, et al. The Efficacy of Infliximab Monotherapy versus Infliximab-Azathioprine Sequential Treatment in Crohn's Disease: Experience from a Tertiary Medical Center in China. BioMed Res Int. 2016;2016.

222. Zhou Y, He H, Wang P, Zhang T, Lin M, Wang H, et al. Infliximab for the treatment of Crohn's disease: Efficacy and safety in a Chinese single-center retrospective study. Eur J Gastroenterol Hepatol. 2015;27(11):1270-5.

223. Zorzi F, Zuzzi S, Onali S, Calabrese E, Condino G, Petruzziello C, et al. Efficacy and safety of infliximab and adalimumab in Crohn's disease: A single centre study. Aliment Pharmacol Ther. 2012;35(12):1397-407.

**Supplemental Table 1: studies excluded (with reasons)**

| **Studies included for detailed review** | **278** |
| --- | --- |
|  |  |
| *Exclusion reason* | *Number of refs excluded* |
| Not full study (e.g. conference abstract, letter, comment, protocol) | 1.406 |
| Case report or case series | 1.037 |
| Not IBD (e.g. psoriasis, rheumatoid arthritis) | 1.014 |
| Not intervention of interest (e.g. azathioprine, lenalidomide, apheresis) | 1.026 |
| Other study group (e.g. children) | 699 |
| In vitro research / animal study | 842 |
| Not outcome of interest (e.g. cancer risk, PROM, radiology) | 1563 |
| Not available in English | 106 |
| Review or meta-analysis | 2.966 |
| Manually deduplicated | 112 |
| **Total** | **10.771** |

Supplemental Table 2: Individual characteristics of excluded studies (ordered alphabetically by author, by year of publication, alphabetically by journal name, by page number)

| **Study** | **Reason for exclusion** |
| --- | --- |
| Adar, 2014 | Only reported adverse events, but no analysis of patient sex |
| Adedokun, 2014 | Reported endoscopic results and adverse events, but no analysis of patient sex |
| Allez, 2010 | Only reported adverse events, but no analysis of patient sex |
| Amiot, 2016 | Only reported adverse events, but no analysis of patient sex |
| Amiot, 2017 | Only reported adverse events, but no analysis of patient sex |
| Ampuero, 2016 | Reported endoscopic results and adverse events, but no analysis of patient sex |
| Appau, 2008 | Only reported adverse events, but no analysis of patient sex |
| Arguelles-Arias, 2017 | Only reported adverse events, but no analysis of patient sex |
| Armuzzi, 2013 | Reported endoscopic results and adverse events, but no analysis of patient sex |
| Baert, 2014 | Only reported adverse events, but no analysis of patient sex |
| Baert, 2010 | Reported endoscopic results and adverse events, but no analysis of patient sex |
| Balint, 2016 | Reported endoscopic results and adverse events, but no analysis of patient sex |
| Barber, 2016 | Reported endoscopic results and adverse events, but no analysis of patient sex |
| Battat, 2017 | Reported endoscopic results and adverse events, but no analysis of patient sex |
| Battat, 2019 | Reported endoscopic results and adverse events, but no analysis of patient sex |
| Bau, 2017 | Only reported adverse events, but no analysis of patient sex |
| Ben-Horin, 2015 | Reported endoscopic results and adverse events, but no analysis of patient sex |
| Ben-Horin, 2018 | Only reported adverse events, but no analysis of patient sex |
| Bernardo, 2019 | Reported endoscopic results and adverse events, but no analysis of patient sex |
| Bjorkesten, 2011 | Reported endoscopic results and adverse events, but no analysis of patient sex |
| Bjorkesten, 2013 | Reported endoscopic results and adverse events, but no analysis of patient sex |
| Boktor, 2016 | Reported endoscopic results and adverse events, but no analysis of patient sex |
| Bosca-Watts, 2016 | Reported endoscopic results and adverse events, but no analysis of patient sex |
| Bossuyt, 2019 | Reported endoscopic results and adverse events, but no analysis of patient sex |
| Bouguen, 2015 | Only reported adverse events, but no analysis of patient sex |
| Bouguen, 2014 (CGH) | Reported endoscopic results and adverse events, but no analysis of patient sex |
| Bouguen, 2014 (IBD) | Reported endoscopic results and adverse events, but no analysis of patient sex |
| Brandse, 2015 | Only reported adverse events, but no analysis of patient sex |
| Buer, 2019 | Only reported adverse events, but no analysis of patient sex |
| Castiglione, 2013 | Reported endoscopic results and adverse events, but no analysis of patient sex |
| Castiglione, 2017 | Reported endoscopic results and adverse events, but no analysis of patient sex |
| Caviglia, 2007 | Only reported adverse events, but no analysis of patient sex |
| Chang, 2014 | Only reported adverse events, but no analysis of patient sex |
| Chaparro, 2012 | Only reported adverse events, but no analysis of patient sex |
| Chaparro, 2018 | Only reported adverse events, but no analysis of patient sex |
| Chiba, 2017 | Reported endoscopic results and adverse events, but no analysis of patient sex |
| Choi, 2016 | Only reported adverse events, but no analysis of patient sex |
| Christensen, 2017 | Reported endoscopic results and adverse events, but no analysis of patient sex |
| Christensen, 2018 | Reported endoscopic results and adverse events, but no analysis of patient sex |
| Christensen, 2019 | Only reported adverse events, but no analysis of patient sex |
| Collins, 2017 | Reported endoscopic results and adverse events, but no analysis of patient sex |
| Colombel, 2009 | Only reported adverse events, but no analysis of patient sex |
| Colombel, 2010 | Reported endoscopic results and adverse events, but no analysis of patient sex |
| Colombel, 2014 | Reported endoscopic results and adverse events, but no analysis of patient sex |
| Colombel, 2014 | Reported endoscopic results and adverse events, but no analysis of patient sex |
| Colombel, 2014 | Reported endoscopic results and adverse events, but no analysis of patient sex |
| Colombel, 2015 | Not all patients treated with biological and/or no sub-analysis of this subgroup |
| Colombel, 2017 | Reported endoscopic results and adverse events, but no analysis of patient sex |
| Colombel, 2018 | Reported endoscopic results and adverse events, but no analysis of patient sex |
| Cotter, 2014 | Reported endoscopic results and adverse events, but no analysis of patient sex |
| Dai, 2014 | Reported endoscopic results and adverse events, but no analysis of patient sex |
| de Barcelos, 2017 | Reported endoscopic results and adverse events, but no analysis of patient sex |
| de Bruyn, 2014 | Reported endoscopic results and adverse events, but no analysis of patient sex |
| de Cruz, 2015 | Reported endoscopic results and adverse events, but no analysis of patient sex |
| de Silva, 2012 | Reported endoscopic results and adverse events, but no analysis of patient sex |
| de Vos, 2012 | Reported endoscopic results and adverse events, but no analysis of patient sex |
| de Vos, 2013 | Reported endoscopic results and adverse events, but no analysis of patient sex |
| d’Haens, 1999 | Reported endoscopic results and adverse events, but no analysis of patient sex |
| d'Haens, 2017 (Gastroenterology) | Reported endoscopic results and adverse events, but no analysis of patient sex |
| d’Haens, 2017 (JCC) | Not all patients treated with biological and/or no sub-analysis of this subgroup |
| diDomenicantonio, 2018 | Only reported adverse events, but no analysis of patient sex |
| Domenech, 2010 | Only reported adverse events, but no analysis of patient sex |
| Drobne, 2018 | Only reported adverse events, but no analysis of patient sex |
| Drobne, 2019 | Reported endoscopic results and adverse events, but no analysis of patient sex |
| Echarri, 2015 | Reported endoscopic results and adverse events, but no analysis of patient sex |
| Farkas, 2014 | Reported endoscopic results and adverse events, but no analysis of patient sex |
| Feagan, 2005 | Reported endoscopic results and adverse events, but no analysis of patient sex |
| Feagan, 2013 | Reported endoscopic results and adverse events, but no analysis of patient sex |
| Feng, 2017 | Reported endoscopic results and adverse events, but no analysis of patient sex |
| Fernandez-Blanco, 2018 | Reported endoscopic results and adverse events, but no analysis of patient sex |
| Ferrante, 2013 | Reported endoscopic results and adverse events, but no analysis of patient sex |
| Fukushima, 2018 | Reported endoscopic results and adverse events, but no analysis of patient sex |
| Geboes, 2005 | Reported endoscopic results and adverse events, but no analysis of patient sex |
| Gecse, 2016 | Only reported adverse events, but no analysis of patient sex |
| Godat, 2018 | Not all patients treated with biological and/or no sub-analysis of this subgroup |
| Goll, 2017 | Only reported adverse events, but no analysis of patient sex |
| Gonci, 2017 | Only reported adverse events, but no analysis of patient sex |
| Greener, 2018 | Reported endoscopic results and adverse events, but no analysis of patient sex |
| Greenup, 2017 | Reported endoscopic results and adverse events, but no analysis of patient sex |
| Guerra-Veloz, 2018 | Only reported adverse events, but no analysis of patient sex |
| Hall, 2014 | Reported endoscopic results and adverse events, but no analysis of patient sex |
| Hamzaoglu, 2010 | Only reported adverse events, but no analysis of patient sex |
| Hansen, 2007 | Only reported adverse events, but no analysis of patient sex |
| Harris, 2016 | Reported endoscopic results and adverse events, but no analysis of patient sex |
| Hebuterne, 2013 | Reported endoscopic results and adverse events, but no analysis of patient sex |
| Hendler, 2015 | Only reported adverse events, but no analysis of patient sex |
| Hibi, 2016 | Reported endoscopic results and adverse events, but no analysis of patient sex |
| Hibi, 2017 | Only reported adverse events, but no analysis of patient sex |
| Hinojosa, 2007 | Only reported adverse events, but no analysis of patient sex |
| Iborra, 2017 | Reported endoscopic results and adverse events, but no analysis of patient sex |
| Imaeda, 2014 | Reported endoscopic results and adverse events, but no analysis of patient sex |
| Jarnerot, 2005 | Reported endoscopic results and adverse events, but no analysis of patient sex |
| Jharap, 2015 | Reported endoscopic results and adverse events, but no analysis of patient sex |
| Jung, 2015 | Reported endoscopic results and adverse events, but no analysis of patient sex |
| Jurgens, 2011 | Reported endoscopic results and adverse events, but no analysis of patient sex |
| Kamat, 2019 | Only reported adverse events, but no analysis of patient sex |
| Kaniewska, 2017 | Reported endoscopic results and adverse events, but no analysis of patient sex |
| Kim, 2019 | Only reported adverse events, but no analysis of patient sex |
| Kiss, 2013 | Combined outcome measure of clinical efficacy and adverse events |
| Kolar, 2016 | Reported endoscopic results and adverse events, but no analysis of patient sex |
| Kolar, 2017 | Reported endoscopic results and adverse events, but no analysis of patient sex |
| Kopylov, 2017 | Only reported adverse events, but no analysis of patient sex |
| Kopylov, 2018 | Only reported adverse events, but no analysis of patient sex |
| Kopylov, 2019 | Only reported adverse events, but no analysis of patient sex |
| Kotze, 2015 (Intestinal Res) | Reported endoscopic results and adverse events, but no analysis of patient sex |
| Kotze, 2015 (JCC) | Reported endoscopic results and adverse events, but no analysis of patient sex |
| Kulaylat, 2017 | Only reported adverse events, but no analysis of patient sex |
| Kurnool, 2018 | Reported endoscopic results and adverse events, but no analysis of patient sex |
| Laharie, 2013 | Reported endoscopic results and adverse events, but no analysis of patient sex |
| Lee, 2013 | Reported endoscopic results and adverse events, but no analysis of patient sex |
| Lees, 2007 | Only reported adverse events, but no analysis of patient sex |
| Lees, 2009 | Only reported adverse events, but no analysis of patient sex |
| Lehtola, 2016 | Reported endoscopic results and adverse events, but no analysis of patient sex |
| Lemann, 2006 | Reported endoscopic results and adverse events, but no analysis of patient sex |
| Lenti, 2018 | Only reported adverse events, but no analysis of patient sex |
| Leung, 2017 | Reported endoscopic results and adverse events, but no analysis of patient sex |
| Li, 2012 | Reported endoscopic results and adverse events, but no analysis of patient sex |
| Lichtiger, 2010 | Only reported adverse events, but no analysis of patient sex |
| Liefferinckx, 2019 | Reported endoscopic results and adverse events, but no analysis of patient sex |
| Liu, 2015 | Only reported adverse events, but no analysis of patient sex |
| Lobaton, 2015 | Only reported adverse events, but no analysis of patient sex |
| Loftus, 2016 | Only reported adverse events, but no analysis of patient sex |
| Lopez-Sanroman, 2017 | Reported endoscopic results and adverse events, but no analysis of patient sex |
| Lykowska-Szuber, 2016 | Manuscript could not be retrieved |
| Ma, 2017 (APT) | Reported endoscopic results and adverse events, but no analysis of patient sex |
| Ma, 2017 (IBD) | Reported endoscopic results and adverse events, but no analysis of patient sex |
| Magro, 2017 | Reported endoscopic results and adverse events, but no analysis of patient sex |
| Mao, 2017 | Reported endoscopic results and adverse events, but no analysis of patient sex |
| Marehbian, 2009 | Only reported adverse events, but no analysis of patient sex |
| Martineau, 2017 | Only reported adverse events, but no analysis of patient sex |
| Matsumoto, 2016 | Reported endoscopic results and adverse events, but no analysis of patient sex |
| Meyer, 2019 | Only reported adverse events, but no analysis of patient sex |
| Miheller, 2009 | Only reported adverse events, but no analysis of patient sex |
| Molander, 2013 | Reported endoscopic results and adverse events, but no analysis of patient sex |
| Moon, 2015 | Only reported adverse events, but no analysis of patient sex |
| Mortensen, 2011 | Reported endoscopic results and adverse events, but no analysis of patient sex |
| Motoya, 2019 | Reported endoscopic results and adverse events, but no analysis of patient sex |
| Munoz-Villafranca, 2018 | Reported endoscopic results and adverse events, but no analysis of patient sex |
| Nakarai, 2014 | Reported endoscopic results and adverse events, but no analysis of patient sex |
| Narula, 2016 | Only reported adverse events, but no analysis of patient sex |
| Noman, 2017 | Reported endoscopic results and adverse events, but no analysis of patient sex |
| Nuki, 2016 | Only reported adverse events, but no analysis of patient sex |
| O’Connell, 2018 | Only reported adverse events, but no analysis of patient sex |
| O’Donnell, 2011 | Only reported adverse events, but no analysis of patient sex |
| Ogata, 2016 | Only reported adverse events, but no analysis of patient sex |
| Ordas, 2014 | Reported endoscopic results and adverse events, but no analysis of patient sex |
| Orlandini, 2018 | Only reported adverse events, but no analysis of patient sex |
| Osterman, 2015 | Only reported adverse events, but no analysis of patient sex |
| Panaccione, 2011 | Only reported adverse events, but no analysis of patient sex |
| Panaccione, 2014 | Reported endoscopic results and adverse events, but no analysis of patient sex |
| Pellet, 2019 | Only reported adverse events, but no analysis of patient sex |
| Peyrin-Biroulet, 2014 | Reported endoscopic results and adverse events, but no analysis of patient sex |
| Pouillon, 2018 | Reported endoscopic results and adverse events, but no analysis of patient sex |
| Raimundo Fernandes, 2016 | Reported endoscopic results and adverse events, but no analysis of patient sex |
| Regueiro, 2016 | Reported endoscopic results and adverse events, but no analysis of patient sex |
| Reinisch, 2013 | Reported endoscopic results and adverse events, but no analysis of patient sex |
| Reinisch, 2015 | Reported endoscopic results and adverse events, but no analysis of patient sex |
| Reinisch, 2017 | Reported endoscopic results and adverse events, but no analysis of patient sex |
| Reinisch, 2019 | Reported endoscopic results and adverse events, but no analysis of patient sex |
| Robbins, 2017 | Reported endoscopic results and adverse events, but no analysis of patient sex |
| Rutgeerts, 1999 | Only reported adverse events, but no analysis of patient sex |
| Rutgeerts, 2004 | Reported endoscopic results and adverse events, but no analysis of patient sex |
| Rutgeerts, 2006 | Reported endoscopic results and adverse events, but no analysis of patient sex |
| Rutgeerts, 2012 | Reported endoscopic results and adverse events, but no analysis of patient sex |
| Rutgeerts, 2018 | Reported endoscopic results and adverse events, but no analysis of patient sex |
| Saigusa, 2016 | Reported endoscopic results and adverse events, but no analysis of patient sex |
| Saito, 2014 | Reported endoscopic results and adverse events, but no analysis of patient sex |
| Samaan, 2017 | Reported endoscopic results and adverse events, but no analysis of patient sex |
| Sandborn, 2012 | Reported endoscopic results and adverse events, but no analysis of patient sex |
| Sandborn, 2013 | Reported endoscopic results and adverse events, but no analysis of patient sex |
| Sandborn, 2014 (APT) | Reported endoscopic results and adverse events, but no analysis of patient sex |
| Sandborn, 2014 (Gastroenterology) | Only reported adverse events, but no analysis of patient sex |
| Sandborn, 2018 | Only reported adverse events, but no analysis of patient sex |
| Sandborn, 2019 | Reported endoscopic results and adverse events, but no analysis of patient sex |
| Sartini, 2019 | Combined outcome measure of clinical efficacy and adverse events |
| Savarino, 2013 | Reported endoscopic results and adverse events, but no analysis of patient sex |
| Schnitzler, 2009 | Reported endoscopic results and adverse events, but no analysis of patient sex |
| Schoepfer, 2010 | Only reported adverse events, but no analysis of patient sex |
| Seah, 2018 | Reported endoscopic results and adverse events, but no analysis of patient sex |
| Seo, 2014 | Reported endoscopic results and adverse events, but no analysis of patient sex |
| Seow, 2010 | Reported endoscopic results and adverse events, but no analysis of patient sex |
| Shmidt, 2018 | Reported endoscopic results and adverse events, but no analysis of patient sex |
| Singh, 2016 (APT) | Only reported adverse events, but no analysis of patient sex |
| Singh, 2016 (CGH) | Only reported adverse events, but no analysis of patient sex |
| Singh, 2018 | Reported endoscopic results and adverse events, but no analysis of patient sex |
| Sorrentino, 2012 | Reported endoscopic results and adverse events, but no analysis of patient sex |
| Subramaniam, 2014 | Reported endoscopic results and adverse events, but no analysis of patient sex |
| Suzuki, 2014 | Reported endoscopic results and adverse events, but no analysis of patient sex |
| Suzuki, 2017 | Reported endoscopic results and adverse events, but no analysis of patient sex |
| Swoger, 2010 | Reported endoscopic results and adverse events, but no analysis of patient sex |
| Taxonera, 2019 | Not all patients treated with biological and/or no sub-analysis of this subgroup |
| Telesco, 2018 | Reported endoscopic results and adverse events, but no analysis of patient sex |
| Tomecki, 2004 | Manuscript could not be retrieved |
| Tursi, 2013 | Manuscript could not be retrieved |
| Tursi, 2014 (Eur J Intern Med p485) | Reported endoscopic results and adverse events, but no analysis of patient sex |
| Tursi, 2014 (Eur J Intern Med p757) | Reported endoscopic results and adverse events, but no analysis of patient sex |
| Tursi, 2017 | Reported endoscopic results and adverse events, but no analysis of patient sex |
| Tursi, 2018 | Reported endoscopic results and adverse events, but no analysis of patient sex |
| Vadan, 2007 | Manuscript could not be retrieved |
| van Assche, 2008 | Reported endoscopic results and adverse events, but no analysis of patient sex |
| Varvarynets, 2018 | Manuscript could not be retrieved |
| Vavricka, 2011 | Only reported adverse events, but no analysis of patient sex |
| Vavricka, 2017 | Only reported adverse events, but no analysis of patient sex |
| Verstockt, 2019 | Reported endoscopic results and adverse events, but no analysis of patient sex |
| Vivio, 2016 | Reported endoscopic results and adverse events, but no analysis of patient sex |
| Watanabe, 2014 | Only reported adverse events, but no analysis of patient sex |
| Weaver, 2019 | Reported endoscopic results and adverse events, but no analysis of patient sex |
| Wils, 2018 | Reported endoscopic results and adverse events, but no analysis of patient sex |
| Wolf, 2014 | Reported endoscopic results and adverse events, but no analysis of patient sex |
| Wright, 2018 | Reported endoscopic results and adverse events, but no analysis of patient sex |
| Wu, 2016 | Reported endoscopic results and adverse events, but no analysis of patient sex |
| Yajnik, 2017 | Only reported adverse events, but no analysis of patient sex |
| Yamada, 2018 | Reported endoscopic results and adverse events, but no analysis of patient sex |
| Yarur, 2016 (Gut) | Reported endoscopic results and adverse events, but no analysis of patient sex |
| Yarur, 2016 (IBD) | Reported endoscopic results and adverse events, but no analysis of patient sex |
| Yarur, 2019 | Reported endoscopic results and adverse events, but no analysis of patient sex |
| Ylisaukko-oja, 2018 | Reported endoscopic results and adverse events, but no analysis of patient sex |
| Yoshida, 2012 | Reported endoscopic results and adverse events, but no analysis of patient sex |
| Yu, 2016 | Reported endoscopic results and adverse events, but no analysis of patient sex |
| Zabana, 2010 | Only reported adverse events, but no analysis of patient sex |
| Zhang, 2016 | Reported endoscopic results and adverse events, but no analysis of patient sex |
| Zhou, 2015 | Only reported adverse events, but no analysis of patient sex |
| Zorzi, 2012 | Only reported adverse events, but no analysis of patient sex |

**Supplemental Table 3a: Quality assessments.** Cohort studies assessed using Newcastle-Ottawa Scale Sorted, by outcome (efficacy studies, then adverse event studies) and alphabetically by author name.

| Author, year | Cohort selection | | | | Comparability | Outcome | | | Total score |
| --- | --- | --- | --- | --- | --- | --- | --- | --- | --- |
|  | Selection of exposed cohort | Selection of non-exposed | Ascertainment of exposure | Outcome not present at start | Comparability of cohorts | Outcome assessment | Sufficient follow-up | Adequacy of follow-up |  |
| Efficacy studies | | | | | | | | | |
| Arias, 2015 | 1 | 1 | 1 | 1 | 2 | 0 | 1 | 0 | 7 |
| Armuzzi, 2013 | 1 | 1 | 1 | 1 | 2 | 0 | 1 | 0 | 7 |
| Beigel, 2014 | 0 | 1 | 1 | 1 | 2 | 0 | 1 | 0 | 6 |
| Brandse, 2015 | 1 | 1 | 1 | 1 | 0 | 0 | 1 | 1 | 6 |
| Crowell, 2018 | 1 | 1 | 1 | 1 | 0 | 0 | 1 | 0 | 5 |
| Dahlen, 2015 | 1 | 1 | 1 | 1 | 0 | 0 | 1 | 1 | 6 |
| Dreesen, 2018 | 0 | 1 | 1 | 1 | 2 | 0 | 1 | 1 | 7 |
| Dulai, 2016 | 1 | 1 | 1 | 1 | 2 | 0 | 1 | 0 | 7 |
| Farkas, 2016 | 1 | 1 | 1 | 1 | 0 | 0 | 1 | 0 | 5 |
| Fay, 2017 | 0 | 1 | 1 | 1 | 0 | 0 | 1 | 0 | 4 |
| Guidi, 2014 | 1 | 1 | 1 | 1 | 2 | 0 | 1 | 1 | 8 |
| Hall, 2014 | 0 | 1 | 1 | 1 | 0 | 0 | 1 | 0 | 4 |
| Hassan, 2017 | 1 | 1 | 1 | 1 | 2 | 0 | 1 | 1 | 8 |
| Hiraoka, 2018 | 0 | 1 | 1 | 1 | 2 | 0 | 1 | 1 | 7 |
| Juncadella, 2018 | 0 | 1 | 1 | 1 | 2 | 0 | 1 | 1 | 7 |
| Kelly, 2016 | 1 | 1 | 1 | 1 | 2 | 0 | 1 | 1 | 8 |
| Kelly, 2017 | 1 | 1 | 1 | 1 | 2 | 0 | 1 | 1 | 8 |
| Kiss, 2011 | 1 | 1 | 1 | 1 | 2 | 0 | 1 | 0 | 7 |
| Koga, 2018 | 0 | 1 | 1 | 1 | 2 | 0 | 1 | 0 | 6 |
| Kotze, 2018 | 1 | 1 | 1 | 1 | 2 | 0 | 1 | 0 | 7 |
| Kuzela, 2012 | 1 | 1 | 1 | 1 | 0 | 0 | 1 | 0 | 5 |
| Morita, 2017 | 0 | 1 | 1 | 1 | 0 | 0 | 1 | 1 | 5 |
| Narula, 2018 | 1 | 1 | 1 | 1 | 2 | 0 | 1 | 1 | 8 |
| Papaconstantinou, 2017 | 0 | 1 | 1 | 1 | 0 | 0 | 1 | 1 | 5 |
| Papamichael, 2016 | 0 | 1 | 1 | 1 | 2 | 0 | 1 | 1 | 7 |
| Papamichael, 2016 | 0 | 1 | 1 | 1 | 2 | 0 | 1 | 1 | 7 |
| Papamichael, 2018 | 0 | 1 | 1 | 1 | 2 | 0 | 1 | 1 | 7 |
| Paul, 2013 | 1 | 1 | 1 | 1 | 2 | 0 | 1 | 1 | 8 |
| Preda, 2016 | 1 | 1 | 1 | 1 | 2 | 0 | 1 | 0 | 7 |
| Ribaldone, 2018 | 1 | 1 | 1 | 1 | 2 | 0 | 1 | 0 | 7 |
| Rismo_b, 2012 | 0 | 1 | 1 | 1 | 2 | 0 | 1 | 0 | 6 |
| Rismo_a, 2012 | 0 | 1 | 1 | 1 | 2 | 0 | 1 | 0 | 6 |
| Roblin, 2014 | 0 | 1 | 1 | 1 | 2 | 0 | 1 | 1 | 7 |
| Shen, 2018 | 0 | 1 | 1 | 1 | 2 | 0 | 1 | 1 | 7 |
| Thomas, 2014 | 0 | 1 | 1 | 1 | 0 | 0 | 1 | 1 | 5 |
| Ungar, 2016 | 0 | 1 | 1 | 1 | 2 | 0 | 1 | 1 | 7 |
| Yacoub, 2018 | 1 | 1 | 1 | 1 | 2 | 0 | 1 | 1 | 8 |
| Zittan, 2016 | 1 | 1 | 1 | 1 | 2 | 0 | 1 | 1 | 8 |
| Adverse event studies | | | | | | | | | |
| Armuzzi, 2019 | 1 | 1 | 1 | 1 | 2 | 0 | 1 | 1 | 8 |
| Colombel, 2004 | 1 | 1 | 1 | 1 | 0 | 0 | 1 | 0 | 5 |
| d'Haens, 2017 | 0 | 1 | 1 | 1 | 2 | 0 | 1 | 0 | 6 |
| Ducharme, 2010 | 0 | 1 | 1 | 1 | 0 | 0 | 1 | 1 | 5 |
| Fidder, 2009 | 1 | 1 | 1 | 1 | 2 | 0 | 1 | 1 | 8 |
| Gonci, 2017 | 1 | 1 | 1 | 1 | 0 | 0 | 1 | 0 | 5 |
| Greener, 2018 | 1 | 1 | 1 | 1 | 2 | 0 | 1 | 1 | 8 |
| Kiss, 2013 | 0 | 1 | 1 | 1 | 0 | 0 | 1 | 1 | 5 |
| Lie, 2017 | 1 | 1 | 1 | 1 | 2 | 0 | 1 | 1 | 8 |
| Mourad, 2015 | 0 | 1 | 1 | 1 | 0 | 0 | 1 | 1 | 5 |
| Sartini, 2019 | 1 | 1 | 1 | 1 | 0 | 0 | 1 | 0 | 5 |
| Seiderer, 2004 | 0 | 1 | 1 | 1 | 0 | 0 | 1 | 1 | 5 |
| Teriaky, 2014 | 1 | 1 | 1 | 1 | 0 | 0 | 1 | 0 | 5 |
| Zelinkova, 2012 | 1 | 1 | 1 | 1 | 0 | 0 | 1 | 0 | 5 |

**Supplemental Table 3b: Risk of bias assessments.** Post-hoc analyses of randomized trials scored using the Cochrane risk of bias assessment tool. Sorted by outcome (efficacy studies, then adverse event studies) and alphabetically by author name.

| Author, year | Selection bias | Performance bias | Detection bias | Attrition bias | Reporting bias | Overall judgement |
| --- | --- | --- | --- | --- | --- | --- |
| Efficacy studies | | | | | | |
| Bouguen, 2015 | Possible risk | Low risk | Low risk | Low risk | Possible risk | Unclear risk of bias |
| De Cruz, 2015 | Possible risk | High risk | Low risk | Low risk | Possible risk | Unclear risk of bias |
| Watanabe, 2018 | Possible risk | High risk | High risk | Low risk | Low risk | Unclear risk of bias |
| Adverse event studies | | | | | | |
| Colombel, 2017 | Low risk | Low risk | Low risk | Low risk | Low risk | Low risk of bias |
| Colombel, 2018 | Low risk | Low risk | Low risk | Low risk | Low risk | Low risk of bias |
| Feagan, 2018 | Low risk | Low risk | Low risk | Low risk | Low risk | Low risk of bias |
